# Rewiring monocyte glucose metabolism via C-type lectin signaling protects against disseminated candidiasis

**DOI:** 10.1371/journal.ppat.1006632

**Published:** 2017-09-18

**Authors:** Jorge Domínguez-Andrés, Rob J. W. Arts, Rob ter Horst, Mark S. Gresnigt, Sanne P. Smeekens, Jacqueline M. Ratter, Ekta Lachmandas, Lily Boutens, Frank L. van de Veerdonk, Leo A. B. Joosten, Richard A. Notebaart, Carlos Ardavín, Mihai G. Netea

**Affiliations:** 1 Department of Internal Medicine and Radboud Center for Infectious diseases (RCI), Radboud University Nijmegen Medical Centre, Geert Grooteplein 8, Nijmegen, the Netherlands; 2 Departamento de Inmunología y Oncología, Centro Nacional de Biotecnología/CSIC, Darwin 3, Madrid, Spain; 3 Laboratory of Food Microbiology, Wageningen University and Research, Wageningen, The Netherlands; 4 Human Genomics Laboratory, Craiova University of Medicine and Pharmacy, Craiova, Romania; University of Birmingham, UNITED KINGDOM

## Abstract

Monocytes are innate immune cells that play a pivotal role in antifungal immunity, but little is known regarding the cellular metabolic events that regulate their function during infection. Using complementary transcriptomic and immunological studies in human primary monocytes, we show that activation of monocytes by *Candida albicans* yeast and hyphae was accompanied by metabolic rewiring induced through C-type lectin-signaling pathways. We describe that the innate immune responses against *Candida* yeast are energy-demanding processes that lead to the mobilization of intracellular metabolite pools and require induction of glucose metabolism, oxidative phosphorylation and glutaminolysis, while responses to hyphae primarily rely on glycolysis. Experimental models of systemic candidiasis models validated a central role for glucose metabolism in anti-*Candida* immunity, as the impairment of glycolysis led to increased susceptibility in mice. Collectively, these data highlight the importance of understanding the complex network of metabolic responses triggered during infections, and unveil new potential targets for therapeutic approaches against fungal diseases.

## Author summary

Fungal infections are a major health concern for immunocompromised individuals due to the lack of success of the currently available antifungal therapies. Unveiling the metabolic processes involved in the immune function offers a promising opportunity for the development of new therapeutic approaches against these infections. In this report, we describe how changes in monocyte glucose metabolism are crucial for host defense against infections caused by the opportunistic pathogenic yeast *Candida albicans*. We report how the participation of various metabolic routes, such as glycolysis, oxidative phosphorylation and the pentose phosphate pathway, were differentially required after yeast or hyphal exposure, depending on the cellular energy requirements for each response. The proper control of metabolic reprogramming of immune cells was crucial to afford protection against fungal infections *in vivo*.

## Introduction

The immune system is constantly challenged by pathogens, and this requires immune cells to optimize the management of metabolic resources in order to exert their crucial role in host defense. A number of studies have shown how different stimuli induce metabolic reprogramming in immune cells, required for the response against microbial infections [1–3].

The recognition of pathogen-associated molecular patterns (PAMPs) triggers substantial changes in cellular metabolism of immune cells, leading to modulation of the effector functions of these cells. Recent studies led to the understanding that the differential use of carbon and nitrogen sources can subsequently affect the immune response. In this sense, proinflammatory macrophages and neutrophils favor aerobic glycolysis over oxidative phosphorylation [4], anti-inflammatory macrophages rely more on fatty acid oxidation and TCA cycle [5], whereas full T cell activation also requires the induction of mitochondrial ROS [6].

*Candida albicans* is a dimorphic fungus that normally colonizes skin and mucosal surfaces in the majority of the healthy population [7], but in immunocompromised hosts can cause severe life threatening infections [8]. Although the cell wall of both *Candida* yeast and hyphae contains a variety of glucans, mannans and glycoproteins that can be recognized by a wide range of PRRs, the expression of these molecules greatly varies between yeast and hyphal forms, leading to substantial differences in cytokine induction [9]. Monocytes undergo metabolic and functional reprogramming after exposure to β-glucans and *C*. *albicans* yeast, leading to a ‘trained immunity’ functional status characterized by an enhanced cytokine production after secondary stimulation with related or non-related stimuli [10]. In addition, monocytes have been shown to play a crucial role against *C*. *albicans* infection, as the deficiency in this immune cell subset has been related with higher susceptibility to fungal infections both in mice and humans [11,12].

Few data are available regarding the role of cellular metabolism for the immune function of monocytes, especially the impact on antifungal host defense. This prompted us to study the metabolic pathways triggered by *C*. *albicans* in human monocytes after yeast or hyphal stimulation, analyzing the different degree of engagement of the main PRRs involved in *C*. *albicans* recognition with the cellular metabolic changes induced, and to study the influence of these alterations on the cytokine response profiles. We report an association between *C*. *albicans-*specific recognition by C-type lectin receptors (CLRs) and the enhancement of glucose metabolism and aerobic glycolysis in monocytes. These metabolic changes are connected with an enhanced proinflammatory cytokine production in a differential way after yeast or hyphal stimulation. The relevance of these immunometabolic changes was validated *in vivo*, showing that inhibition of glucose metabolism led to impaired cytokine production, lower fungicidal activity, and a higher susceptibility to systemic *C*. *albicans* infection.

## Results

### *C*. *albicans* upregulates glycolysis-related genes

**Fig 1:**
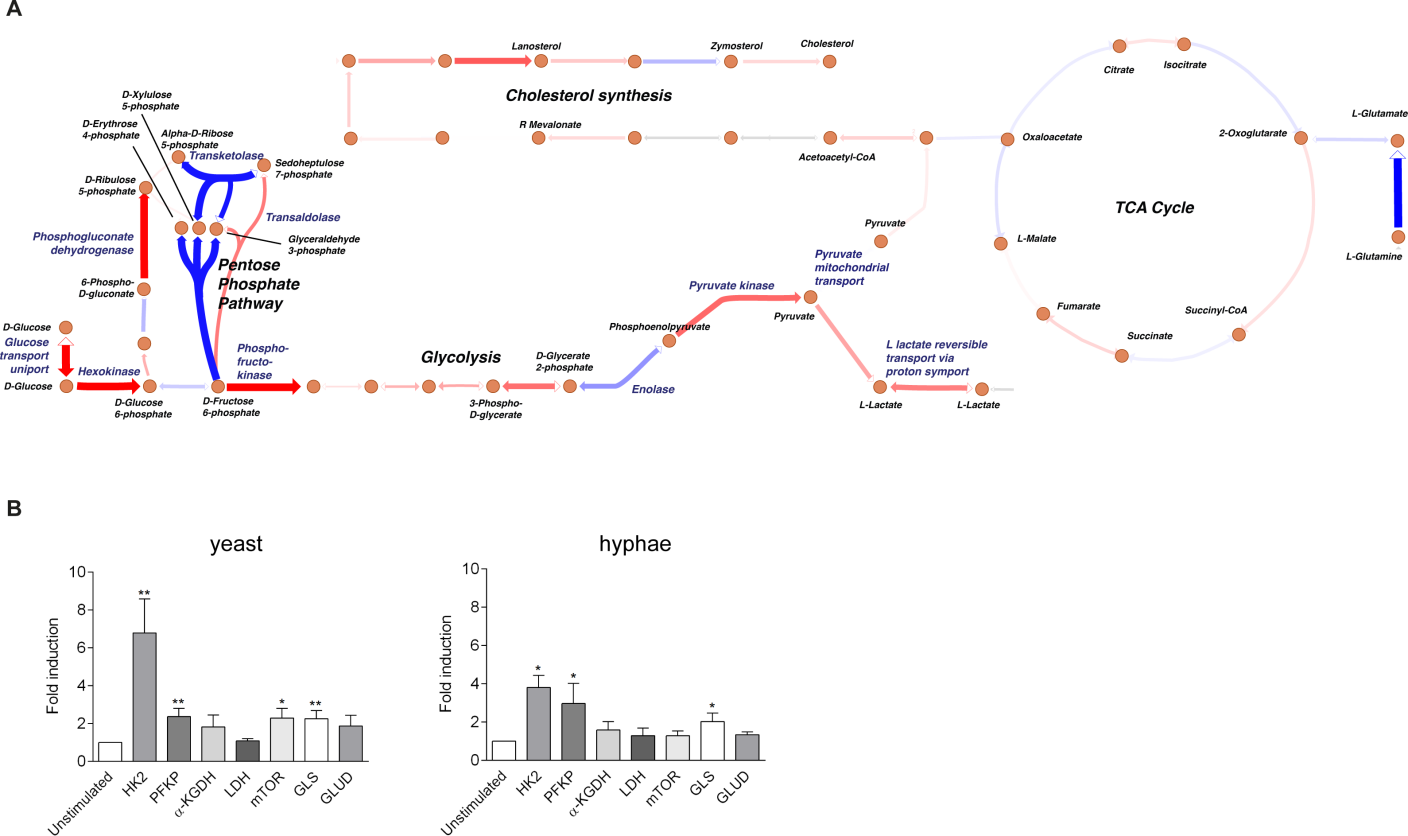
Glycolysis upregulation upon *Candida* stimulation. (A) Schematic pathway map of the gene expression in the main metabolic pathways in PBMCs stimulated with heat-killed *C*. *albicans* yeast 24 h after stimulation. The dots represent metabolites, and the arrows indicate reactions converting these metabolites. For each reaction it is known which enzymes (and thus which genes) are involved in catalyzing the reaction. The arrows marked in red indicate an overall upregulation of genes involved in those reactions in *C*. *albicans* versus RPMI, whereas the blue indicates a downregulation. A darker color indicates a larger change in transcript levels. The complete map stimulation as created by Escher for 4 h and 24 h stimulation is shown in S1 Fig. (B) Fold increase of mRNA expression for the indicated enzymes analyzed by RT-PCR in monocytes 24 h after stimulation with heat-killed *C*. *albicans* conidia or heat-killed *C*. *albicans* hyphae (mean ± SEM, n = 6–9; pooled from 2–3 experiments). *p<0.05, **p<0.01 Wilcoxon signed-rank test. HK2: Hexokinase 2; PFKP: Phosphofructokinase, platelet; α-KGDH: alpha-ketoglutarate dehydrogenase; LDH: Lactate dehydrogenase; mTOR: Mammalian target of rapamycin; GLS: Glutaminase; GLUD: Glutamine dehydrogenase.

Since the molecules expressed in the cell wall of *C*. *albicans* can be recognized by a great variety of receptors that could be involved in the upregulation of different metabolic pathways, we measured genome-wide transcriptional profiles in peripheral blood mononuclear cells (PBMCs) from healthy volunteers upon stimulation with *C*. *albicans* for 4 h and 24 h. Transcriptomic analysis of the genes involved in the main cellular metabolic pathways revealed that the only pathway whose gene expression was consistently upregulated after stimulation was glycolysis, and that this enhancement occurred at 24h, but not at 4h after stimulation (Figs 1A and S1). We validated the upregulation observed in those genes by qPCR in monocytes isolated from healthy volunteers stimulated with heat-killed yeast or hyphae for 24 h finding a significant upregulation of some of the main enzymes involved in glycolysis such as hexokinase (HK) and phosphofructokinase (PFKP). We also found an upregulation of the expression of glutaminase (GLS), an enzyme that allows the entrance of glutamine into tricarboxylic acid (TCA) cycle by converting it into glutamate that is subsequently transformed into α-ketoglutarate (Fig 1B).

### *C*. *albicans* recognition upregulated glycolysis and oxidative phosphorylation in monocytes

**Fig 2:**
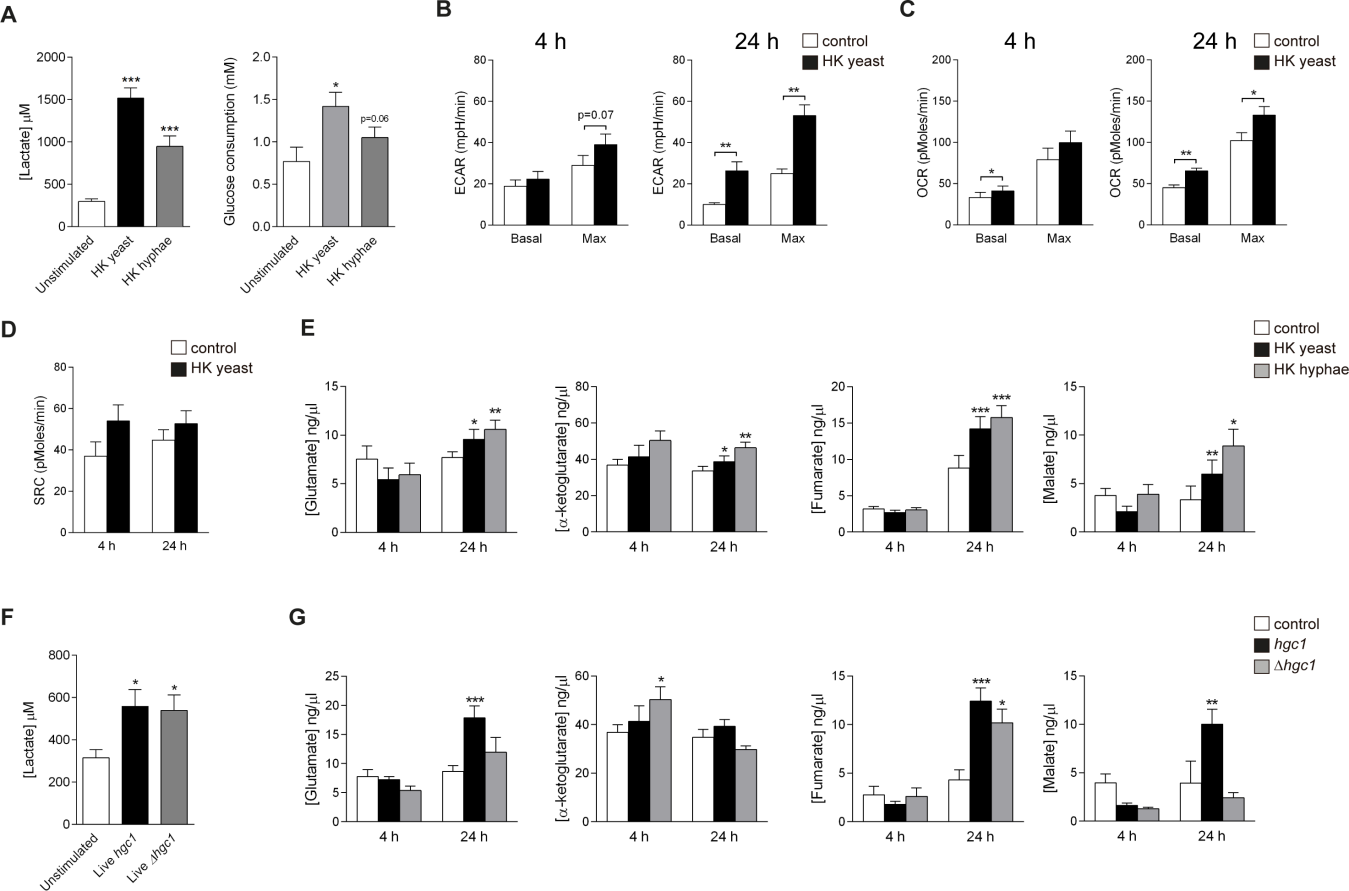
*Candida* stimulation induced glycolysis in human monocytes. (A) Lactate production and glucose consumption by monocytes after overnight stimulation with heat-killed *C*. *albicans* yeast or heat-killed *C*. *albicans* hyphae (mean ± SEM, n = 12 for lactate, n = 6 for glucose; pooled from 2–4 independent experiments). *p<0.05, ***p<0.001 Wilcoxon signed-rank test. (B-D) Basal and maximum extracellular acidification rates (ECAR; B), basal and maximum oxygen consumption rates (OCR; C) or spare respiratory capacity (SRC; D) of monocytes were determined by Seahorse analysis at 4 and 24 h after stimulation with medium or heat-killed *C*. *albicans* yeast (mean ± SEM, n = 6–8; pooled from 2 independent experiments). *p<0.05, **p<0.01 Wilcoxon signed-rank test. (E) Intracellular metabolite levels of monocytes 4 and 24 h after heat-killed *C*. *albicans* yeast or heat-killed *C*. *albicans* hyphae stimulation (mean ± SEM, n = 6–8; pooled from 2 independent experiments). *p<0.05, **p<0.01 Wilcoxon signed-rank test. (F) Lactate production by monocytes after overnight stimulation with live *hgc1* or *Δhgc1 C*. *albicans*. (mean ± SEM, n = 6; pooled from 2 independent experiments). *p<0.05, Wilcoxon signed-rank test. (G) Intracellular metabolite levels of monocytes 4 and 24 h after *hgc1* or *Δhgc1* live *C*. *albicans* stimulation (mean ± SEM, n = 6; pooled from 2 independent experiments). *p<0.05, **p<0.01 Wilcoxon signed-rank test.

The upregulation of the expression of genes involved in glycolysis has been related to a boost of glucose consumption and lactate production [13]. In agreement with these data, we observed a significant increase in the lactate concentrations released in the supernatants of *C*. *albicans*-stimulated monocytes, which was accompanied by an increase in glucose consumption both after yeast and hyphal stimulation (Fig 2A), reflecting the induction of the glycolytic pathway [14]. In line with these data, the increased basal and maximal extracellular acidification rate (ECAR) values measured in these cultures reflected an enhancement of the glycolytic activity of monocytes after *C*. *albicans* yeast stimulation (Fig 2B). Importantly, the oxygen consumption rate (OCR), which is accepted to be an indicator of the oxidative phosphorylation activity [15], was also higher in monocytes that had been stimulated with *C*. *albicans* both for 4 and 24 h (Fig 2C), also reflecting an enhancement of the oxidative mitochondrial activity in *C*. *albicans*-stimulated monocytes. Of note, the OCR/ECAR ratio did not change within the time points measured reflecting a proportional increase of glycolysis and OXPHOS (S2 Fig). In addition to this, *C*. *albicans*-stimulated monocytes showed a slightly increased mitochondrial spare respiratory capacity (SRC), especially 4 h after stimulation (Fig 2D).

Since the stimulation of monocytes with *C*. *albicans* led to an increase in glycolysis and oxidative phosphorylation, we quantified the levels of different metabolites of the TCA cycle. Interestingly, after 4 h stimulation with yeast a slight decrease was observed in the levels of glutamate, fumarate and malate, metabolites than can be synthesized from glutamine [16]. On the other hand, overnight stimulation induced a general increase in the intracellular metabolite levels (Fig 2E), suggesting that within 24 h after stimulation, cells had time to induce an extensive activation of the cellular metabolic machinery and fulfill the energy requirements needed for the functional changes induced by cell activation. Of note, these data correspond to the increases in ECR and OCAR observed 4 and 24 h after stimulation (Fig 2C and 2D), reflecting an enhancement of the cellular metabolic activity after *C*. *albicans* recognition by monocytes. We identified no overall changes in cell numbers after stimulation of cells with *C*. *albicans*, we can thus conclude that cell growth or an enhanced survival of monocytes stimulated with *C*. *albicans* is not the reason of the differences observed in ECAR or OCR.

Heat-killing alters the cell wall structure and exposes antigens and PAMPs on the surface of *C*. *albicans* yeasts and hyphae [17], and we validated the results obtained with heat-killed forms of the fungus by using live yeast and hyphae. In order to distinguish between the metabolic changes induced either by yeast or hyphae, we cultured monocytes with a yeast-locked strain of *C*. *albicans* (*Δhgc1*) or with the hyphae-forming wild-type corresponding strain (*hgc1*), as the *in vivo* culture conditions used stimulate hyphal development from live yeast. As described for heat-killed forms, stimulation with live *C*. *albicans* increased lactate production by human monocytes (Fig 2F). Of note, the increase in lactate measured after stimulation with live fungal forms was lower than with heat-killed forms, most likely due to the effective masking of β–glucan in the cell wall of live yeasts [18]. In line with this hypothesis, the levels of intracellular metabolites 24 h after infection were significantly higher for hyphae-forming live *Candida* than for yeast-locked *Candida* (Fig 2G).

### Glucose metabolism modulates cytokine response after *Candida* stimulation

**Fig 3:**
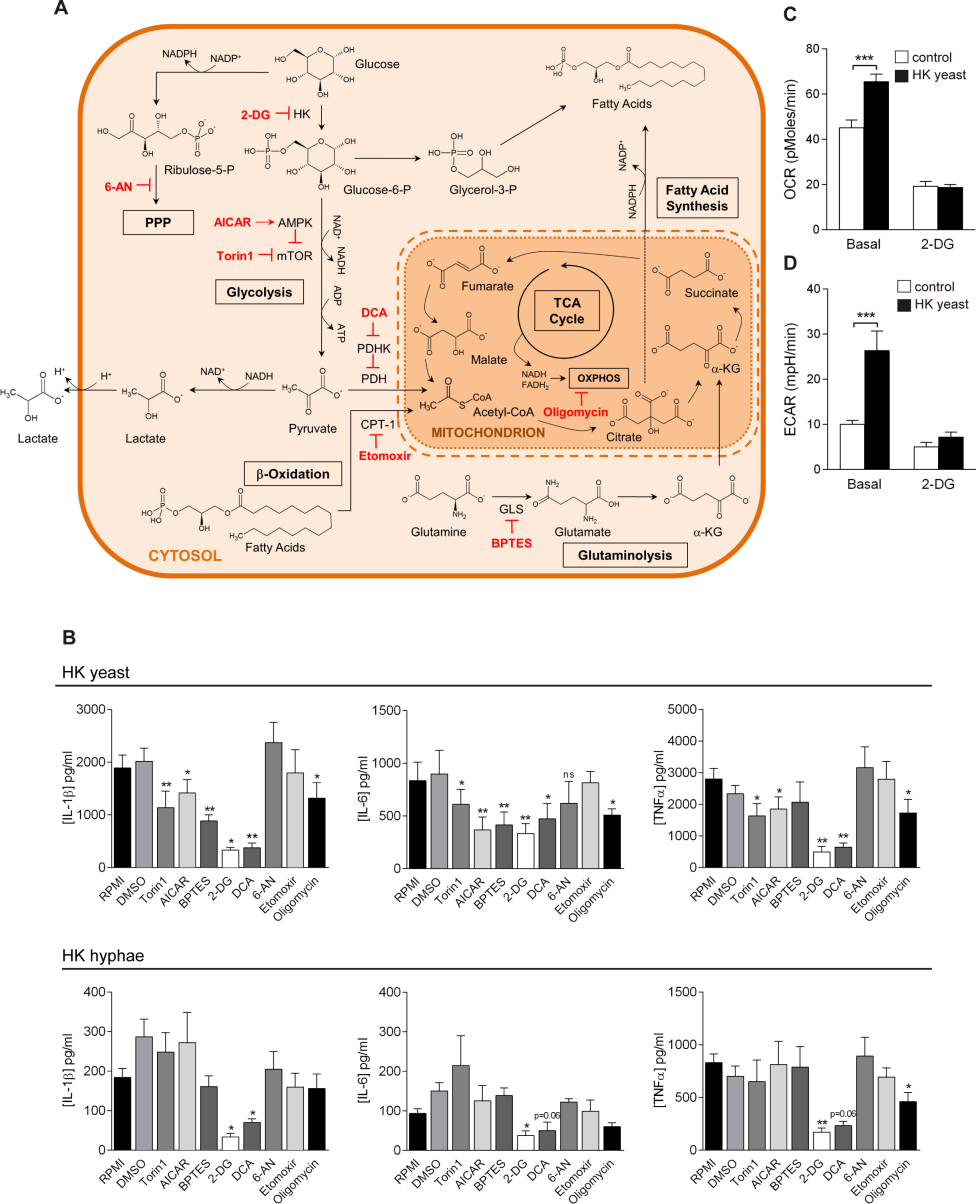
Glycolysis, glutaminolysis and oxidative phosphorylation are involved in cytokine production after *Candida* stimulation. (A) Scheme of the chemical inhibitors used, their target enzymes, and the main intracellular metabolic pathways studied. (B) IL-1β, IL-6 and TNFα production by human monocytes treated with different metabolic inhibitors and stimulated with heat-killed *C*. *albicans* yeast or heat-killed *C*. *albicans* hyphae for 24 h (mean ± SEM, n = 6; pooled from 2 independent experiments). *p<0.05, **p<0.01 Wilcoxon signed-rank test. (C-D) Basal OCR (C) and ECAR (D) of monocytes were determined 24 h after stimulation with medium or heat-killed *C*. *albicans* yeast by extracellular flux measurements in basal conditions of after injection of 2-DG (mean ± SEM, n = 6; pooled from 2 independent experiments). *p<0.05, **p<0.01 Wilcoxon signed-rank test.

Apart from phagocytosis and killing, one of the main effector functions of monocytes and macrophages during *C*. *albicans* infection is the production of proinflammatory cytokines, required for the development of a protective immune response [8]. Therefore, we tested how the specific inhibition of various metabolic pathways affected the proinflammatory cytokine production after stimulation with *C*. *albicans*-yeast and hyphae (Fig 3A). The inhibition of glycolysis with 2-deoxyglucose (2-DG), a competitive inhibitor of hexokinase (HK), or with dichloroacetate (DCA), a compound that skews the glycolytic flux through TCA cycle by reducing the transformation of pyruvate into lactate by enhancing the activity of pyruvate dehydrogenase (PDH), strongly downregulated *C*. *albicans-*induced IL-1β, TNFα and IL-6 production in human monocytes (Fig 3B). Monocyte training with β-glucan, a ligand from *C*. *albicans* cell wall, has been reported to cause a switch from oxidative phosphorylation to aerobic glycolysis via activation of the PI3K-Akt-mTOR axis in human monocytes [19]. We found that inhibition of the mTOR pathway with Torin1 (a direct mTOR inhibitor) or with AICAR (an indirect mTOR inhibitor via AMPK activation) caused a decrease in the cytokine production by human monocytes after stimulation with yeast, but not with hyphae (Fig 3B).

In addition, inhibition of glutaminolysis by BPTES, a selective inhibitor of glutaminase (GLS), also impaired the production of IL-1β and IL-6 in yeast-stimulated monocytes, although to a lower extent (Fig 3B). The inhibition of β-oxidation with etomoxir or the interference of the pentose phosphate pathway with 6-aminonicotinamide (6-AN) did not produce any significant differences in the production of the cytokines measured (Fig 3B). Importantly, impairment of oxidative phosphorylation with oligomycin, an ATP synthase inhibitor, caused a significant decrease in the proinflammatory cytokine production after stimulation with *C*. *albicans* yeast (Fig 3B), and this effect is consistent with the increased OCR reported after *C*. *albicans* stimulation (Fig 2B). We confirmed the importance of glycolysis in these processes as 2-DG treatment of monocytes abolished the differences observed in ECAR and OCR after *C*. *albicans* recognition (Fig 3C and 3D). As a whole, these data suggest that *C*. *albicans* yeast-induced cytokine production in monocytes relied on an mTOR-dependent enhanced glycolysis as well as on an increased oxidative phosphorylation activity of the cells and, to a lesser extent, on glutamine metabolism. In the case of hyphae-induced cytokine production, these data suggest that it mostly relies on glycolysis.

Since Th1 and Th17 responses have been reported to play a protective role in *C*. *albicans* infection [20,21] we also measured how the inhibition of the different metabolic pathways affected the production of Th1/Th17-derived cytokines after *C*. *albicans* stimulation of human PBMCs. We found that the inhibition of glucose metabolism notably impaired IL-17, IL-22, IFNγ and IL-10 production, while glutamine metabolism also played a role in the production of Th17-derived cytokines after yeast but not after hyphal stimulation (S3 Fig). In the case of the anti-inflammatory cytokine IL-10, its production after hyphal stimulation was only significantly affected by glycolysis inhibition, suggesting that the metabolic pathways leading to the production of pro- or anti-inflammatory cytokines might be regulated by different metabolic routes, as already described for macrophages [22].

### C-type lectin-signaling pathways trigger glycolysis after stimulation with *C*. *albicans* yeasts

**Fig 4:**
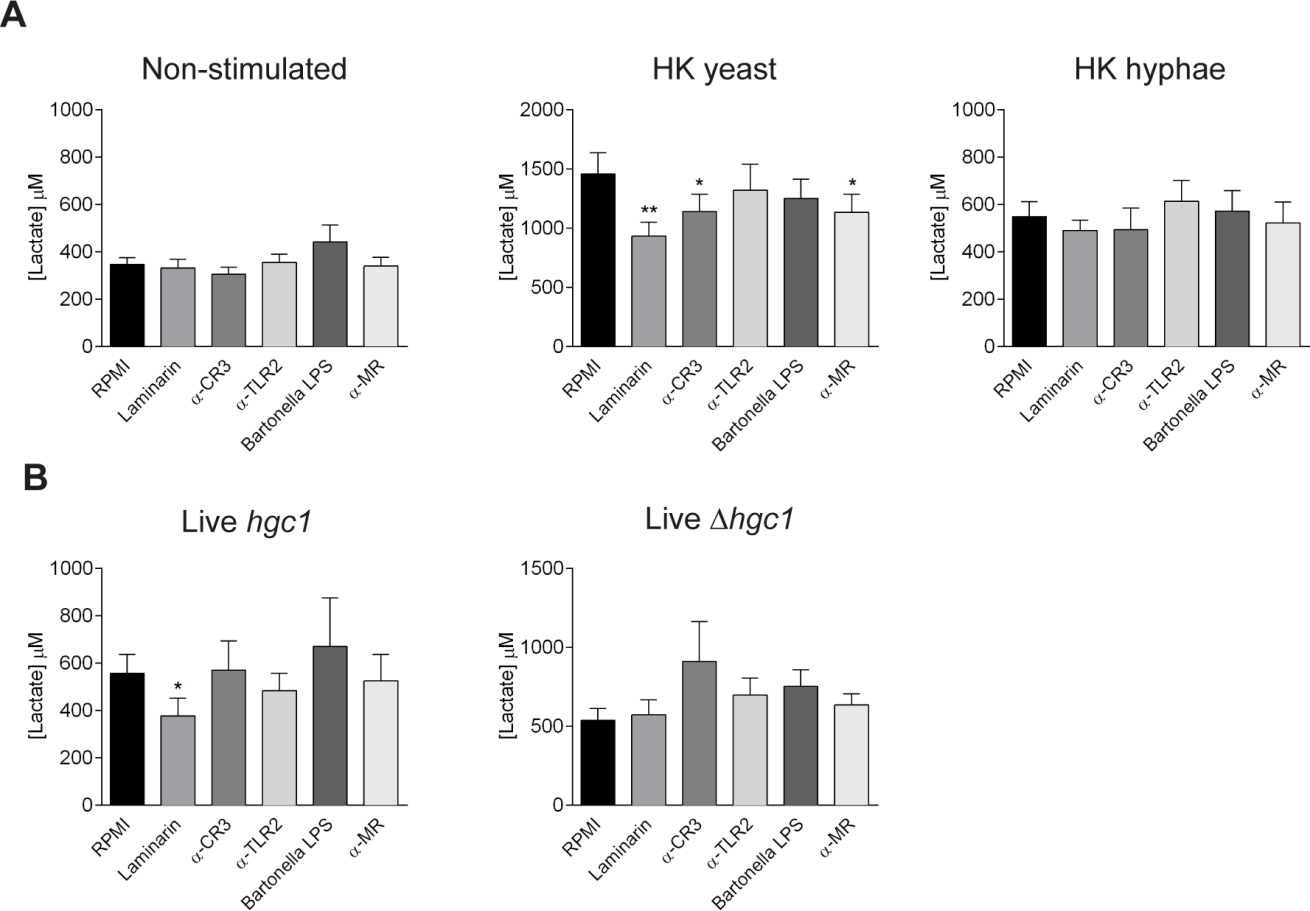
C-type lectins triggered glycolysis after stimulation with *Candida* yeasts. (A-B) Lactate production by human monocytes was measured after blockade of dectin-1, CR3, TLR2, TLR4 and MR and subsequent 24 h stimulation with medium, heat-killed *C*. *albicans* yeast or heat-killed *C*. *albicans* hyphae (A) or *hgc1* or *Δhgc1* live *C*. *albicans* (B) (mean ± SEM, n = 6; pooled from 2 independent experiments). *p< 0.05, **p<0.01 Wilcoxon signed-rank test. Results of stimulation with isotype controls are displayed in S3 Fig.

*C*. *albicans* recognition by human cells is known to rely on a wide series of Pattern Recognition Receptors (PRRs) such as Toll-Like Receptors (TLRs) and C-type lectin receptors (CLRs) [8]. Since *C*. *albicans*-induced cytokine production in human monocytes seemed to be under the control of glycolysis, we wondered which receptors were responsible for triggering the metabolic changes reported. To this aim, we blocked different PRRs involved in *C*. *albicans* recognition and assessed lactate production in cell supernatants after overnight culture. Interestingly, neither the treatment of monocytes with a specific mAb against TLR2, nor the blockade of TLR4 with *Bartonella quintana* LPS, a natural antagonist of this receptor [23] produced any changes in the lactate production triggered by heat-killed *Candida* stimulation, indicating that TLR-derived signaling did not play a role in the induction of glycolysis after *Candida* recognition (Fig 4A). Nevertheless, blockade of C-type lectin receptors with laminarin (a dectin-1 specific antagonist) or with a specific mAb against mannose receptor (MR), caused a significant decrease in the lactate production measured upon stimulation with heat-killed *Candida* yeasts, but not hyphal stimulation. Of note, blockade of CR3, a receptor that has a lectin domain involved in *C*. *albicans* [24] and β-glucan [25] recognition, also led to a decrease in the extracellular lactate levels determined after *C*. *albicans* yeast stimulation (Fig 4A). These results reflect that metabolic changes induced by recognition of *C*. *albicans* by monocytes were mainly driven by CLR-mediated rather than TLR-mediated signaling, in contrast to the metabolic rewiring induced by bacteria [26]. These data also confirmed the differences in the intracellular metabolic requirements triggered after yeast or hyphal recognition.

PRR blockade after monocyte stimulation with live yeast-locked *C*. *albicans* did not produce any significant changes in lactate production except for the case of monocytes with an impaired dectin-1 signaling, which showed a discrete reduction in this readout (Fig 4B). This can be again attributed to the low degree of β-glucan exposure in the cell wall of the live wild-type *C*. *albicans* [27]. The differences seen after live dectin-1 blockade in wild-type *C*. *albicans*-stimulated cells can be explained by the fact that β-glucan, the dectin-1 ligand, is highly exposed in the cell wall of newly-formed hyphae as described by Cheng et al. [18], and further confirmed that yeast and hyphae triggered metabolic changes in human monocytes in a differential fashion.

### ROS production required the participation of glycolysis and the pentose phosphate pathway

**Fig 5:**
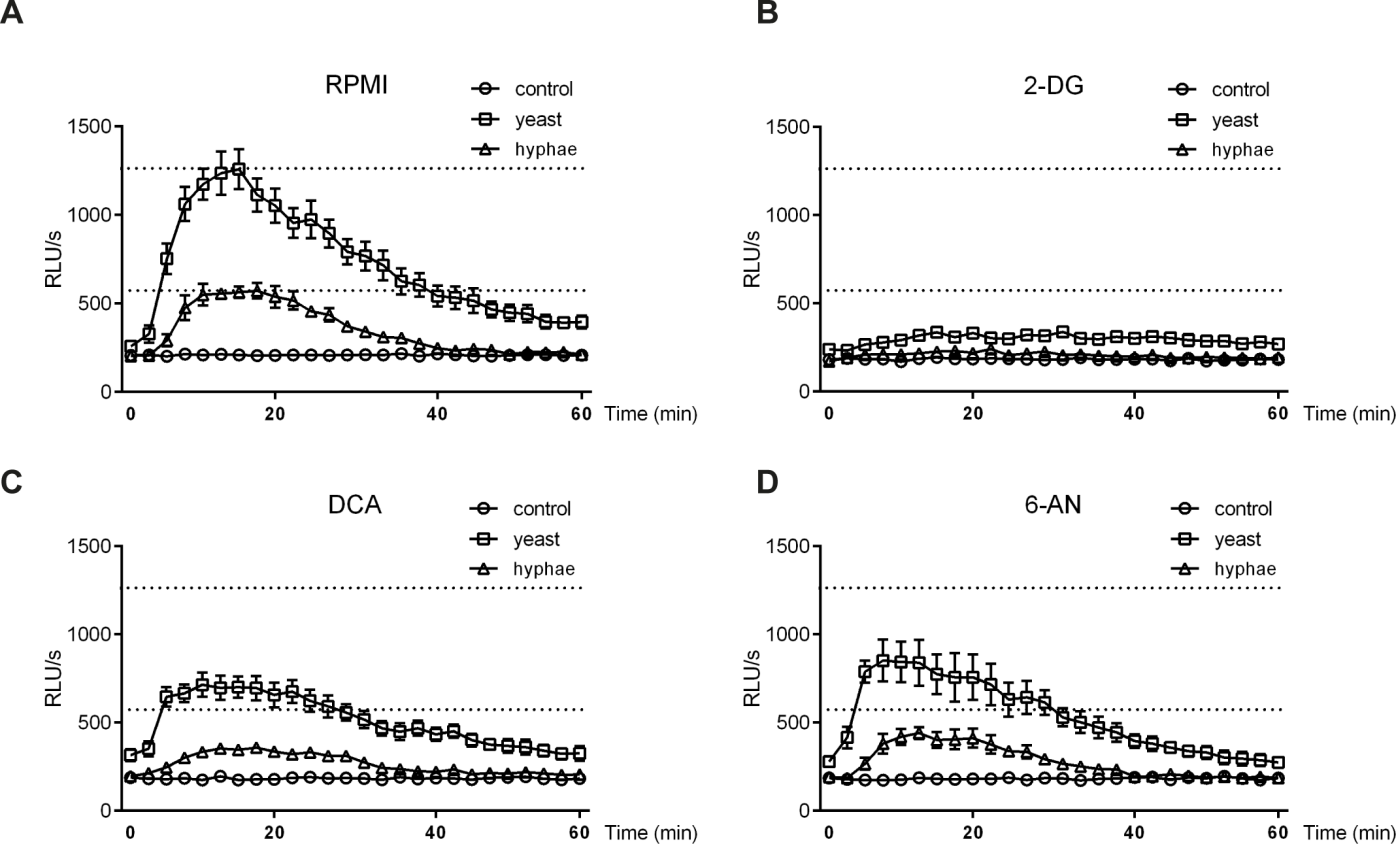
ROS production by monocytes involved glycolysis and the pentose phosphate pathway. (A-D) Human monocytes were treated with medium (A), 2-DG (B), DCA (C) or 6-AN (D) and subsequently stimulated with medium, heat-killed *C*. *albicans* yeast or heat-killed *C*. *albicans* hyphae. Luminescence generated from ROS production was measured every 145 seconds during 60 minutes (n = 4; pooled from 2 independent experiments). Dashed lines show maximum ROS levels reached without inhibitor treatment to be used as a visual reference.

A number of studies have related the production of Reactive Oxygen Species (ROS) with the resolution of *C*. *albicans* infection [8,28]. We hypothesized that ROS induction could be affected by monocyte metabolism after *C*. *albicans* stimulation. Glycolysis inhibition with 2-DG prior to stimulation with yeast or hyphae almost completely abolished ROS production by monocytes (Fig 5B). In line with this, the impairment of the glycolytic routes with DCA treatment also impaired ROS production strongly (Fig 5C). We did not find any other metabolic pathways involved in ROS production after *C*. *albicans* stimulation (S5 Fig), except for the case of the pentose phosphate pathway, for which the specific inhibition of its oxidative branch with 6-AN led to a strong decrease in ROS production (Fig 5D). This can be related to the drop in availability of NADPH, a key factor for the induction of ROS, as already described for LPS-activated macrophages [29]. On the other hand, phagocytosis of *C*. *albicans* yeast was not significantly affected by treatment of monocytes with 2-DG (S6 Fig).

### Inhibition of glucose metabolism impaired *in vivo* responses to *C*. *albicans* infection

**Fig 6:**
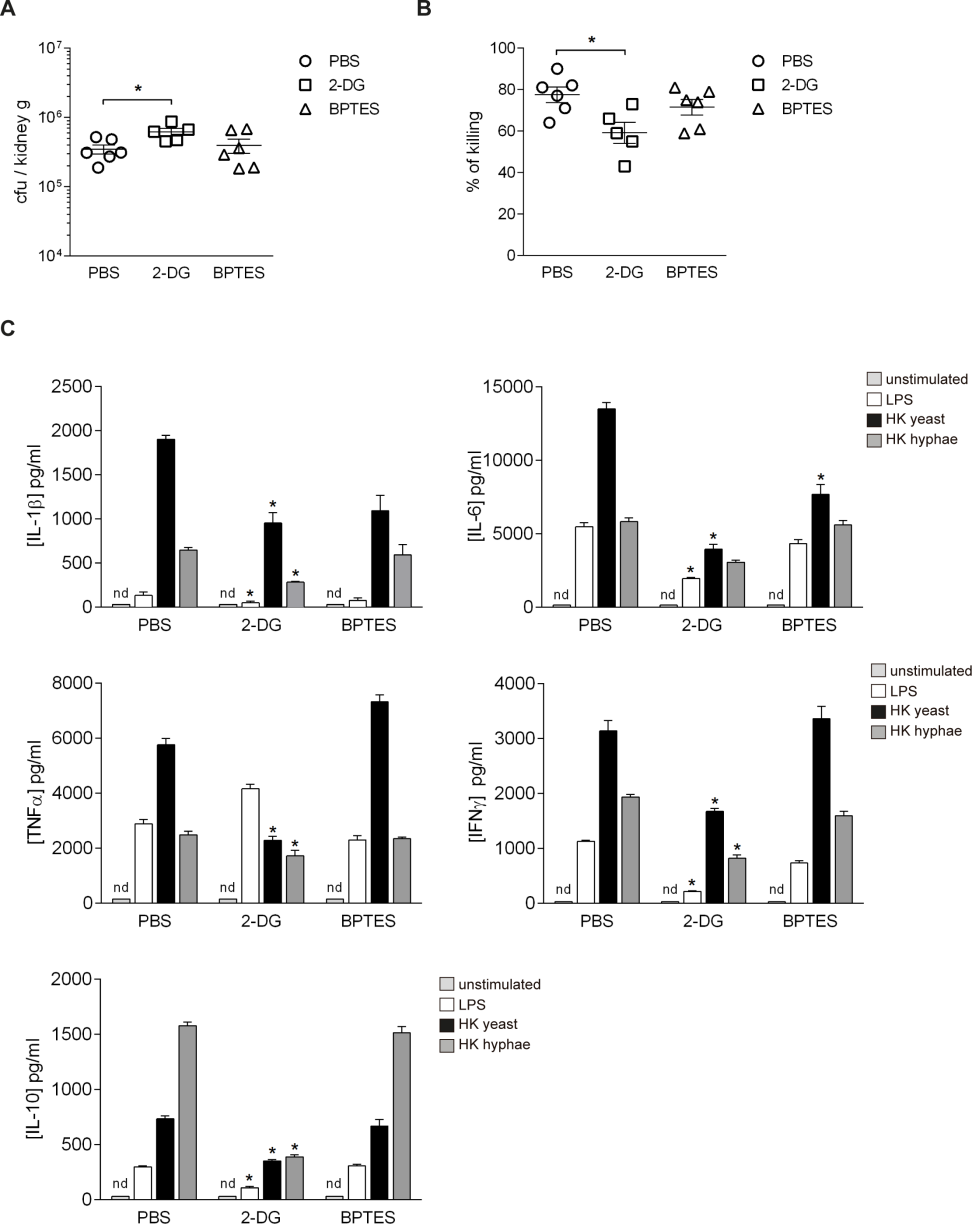
Inhibition of glucose metabolism impaired *in vivo* responses to systemic *C*. *albicans* infection. (A) Fungal burden measured in the kidneys of C57BL/6 mice treated with PBS, 2-DG or BPTES during 5 days after i.v. *C*. *albicans* challenge (mean ± SEM, n = 6; similar results were obtained in 2 independent experiments). *p<0.05, Student’s t test. Each dot represents one mouse. (B) Candidacidal activity of neutrophils isolated from blood of C57BL/6 mice treated with PBS, 2-DG or BPTES during 5 days after i.v. *C*. *albicans* challenge (mean ± SEM, n = 6; similar results were obtained in 2 independent experiments) *p < 0.05, Student’s t test. Each dot represents one mouse. (C) IL-1β, IL-6, IL-10, IFNγ and TNFα production by mouse splenocytes obtained from PBS, 2-DG or BPTES-treated mice 5 days after *C*. *albicans* i.v. infection were measured by ELISA 48 h after *in vitro* restimulation with medium, LPS, heat-killed *C*. *albicans* yeast or heat-killed *C*. *albicans* hyphae (mean ± SEM, n = 6; similar results were obtained in 2 independent experiments) *p<0.05, Student’s t test.

**Fig 7:**
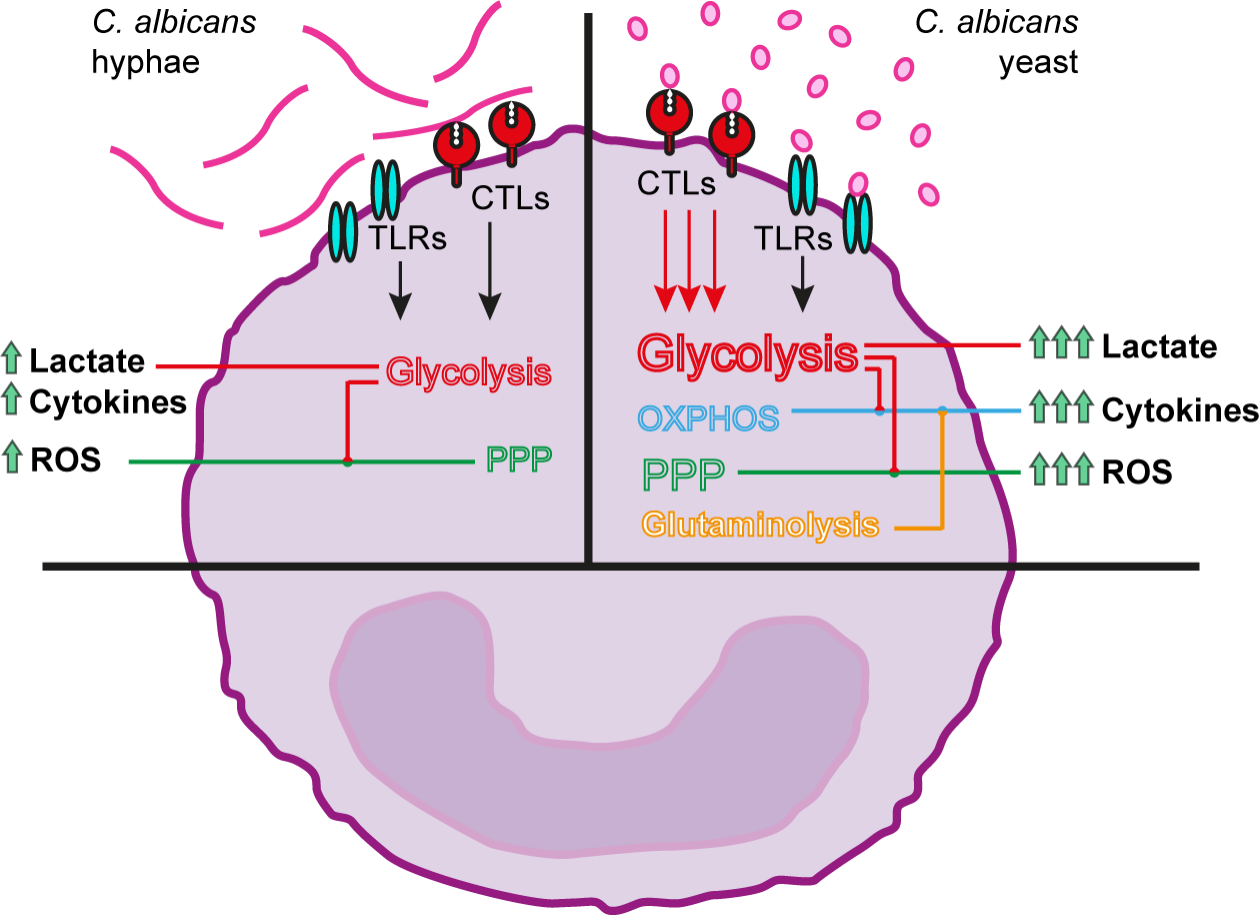
Overview of the immune and metabolic processes taking place in monocytes after systemic *C*. *albicans* recognition. *C*. *albicans* recognition by monocytes triggers a complex network of metabolic pathways that lead to the production of proinflammatory cytokines and ROS, and also the release of lactate to the extracellular space. Heat killing of *C*. *albicans* resulted in higher β-glucan exposure and subsequent dectin-1 recognition while shielding of β-glucans by mannans in hyphae prevents activation of the CTL signaling pathways. While heat-killed hyphae-derived responses mainly rely on the participation of glycolysis and the pentose phosphate pathway, the response to heat-killed yeast is a much more demanding process that requires the participation of glycolysis, oxidative phosphorylation, glutaminolysis and the pentose phosphate pathway through a process driven by an enhanced C-type lectin-derived signaling.

Because the data presented above argue for a crucial role of monocyte glycolysis for antifungal host defense, we wanted to validate these results in an *in vivo* model of systemic *C*. *albicans* infection. In this model, C57BL/6 mice were intravenously injected with a single dose of 10^5^ colony-forming units (CFU) of *C*. *albicans* yeast, causing a disseminated infection [30]. In order to validate the role of glucose and glutamine metabolism *in vivo*, we treated these mice with 2-DG or BPTES prior to systemic *C*. *albicans* intravenous challenge and evaluated the systemic response to infection. Treatment with 2-DG led to a significant increase in the fungal burden measured in the kidneys of these mice 5 days after *C*. *albicans* infection, while BPTES-treated animals had a fungal burden comparable to control individuals (Fig 6A). Of note, one of the mice treated with 2-DG had to be euthanized during the experiment due to the infectious process. We also assessed the candidacidal activity of blood neutrophils, which have been described to be the main effector cells in this model of infection [8], finding a strong impairment of their fungicidal potential in the case of 2-DG treated mice (Fig 6B). Mouse neutrophils treated *in vitro* with 2-DG also had a significantly lower candidacidal activity than control cells (S7 Fig). We also measured cytokine production after *C*. *albicans* restimulation of splenocytes obtained from 2-DG or BPTES-treated mice after the infection, finding a significant reduction in the production capacity of IL-1β, IL-6, IL-10, TNFα and IFNγ in mice treated with 2-DG. Thus, the impairment of glucose metabolism alters the capacity of splenocytes to respond to a secondary *C*. *albicans* stimulation (Fig 6C). In mice treated with BPTES, we found reduced levels of IL-6, which is in agreement with the data obtained from human monocytes. Therefore, while the inhibition of glutamine metabolism seems to have a relatively small effect in systemic antifungal response *in vivo*, these data confirm that glycolysis plays a central role in the induction of an effective anti- *C*. *albicans* host response both *in vitro* and *in vivo* (Fig 7).

## Discussion

*C*. *albicans* is the most important fungal pathogen, and immunotherapeutic approaches to boost antifungal host defense are urgently needed to decrease mortality in systemic candidiasis which currently reaches up to 30–40% [31]. Here we aimed to investigate for the first time the role of cellular metabolism of immune cells for the induction of an effective immune defense against *C*. *albicans*. We observed that *C*. *albicans* yeast and hyphae induce differential rewiring of cellular metabolism: while yeast-stimulated monocytes rely on glycolysis, oxidative phosphorylation and glutaminolysis to mount cytokine responses, monocytes stimulated with hyphae rely mainly on glycolysis. These processes are mediated by fungal recognition by CLRs, but not by TLRs, and glycolysis is crucial for an effective host defense *in vivo* against disseminated candidiasis.

The metabolic circuits triggered in immune cells following pathogen recognition are very complex. Microbial stimuli such as LPS promote glucose conversion into lactate and decrease oxidative phosphorylation in monocytes and macrophages, in a process known as the Warburg effect [4]. However, a recent study demonstrated that induction of the Warburg effect in monocytes is a specific feature of LPS engagement of TLR4, as the engagement of other TLRs by their specific ligands or by complete microorganisms led to a much more complex response, notably a strong increase in the oxidative phosphorylation activity of the cells after stimulation [26]. While several studies investigated the immunometabolic circuits involved in anti-bacterial responses, very few have addressed the role of these pathways in anti-fungal immunity. In this report, we describe differential roles for glucose metabolism, glutamine metabolism, oxidative phosphorylation, and the pentose phosphate pathway in the immune response against *C*. *albicans*.

The increase of glycolysis following microbial stimulation of myeloid cells has been reported in several studies, being usually linked to an enhanced function of mTOR-related pathways [19,32]. Our data confirmed that after *C*. *albicans* recognition, human monocytes underwent an increase not only in their glycolytic activity, as demonstrated by the higher glucose consumption, lactate production and ECAR reported after stimulation, but also in their oxidative phosphorylation capacity and OCR. This enhancement of both aerobic glycolysis and oxidative phosphorylation is reminiscent of the metabolic stimulation induced by other whole microorganisms [16,26], in contrast to purified ligands such as LPS or β-glucan [19,33], reflecting the complex nature of the cellular metabolic networks induced by the engagement of different PRRs.

An important discovery is the difference in induction of cellular metabolism between *C*. *albicans* yeasts and hyphae. The differences in the abundance and the degree of exposure of cell wall components such as β-glucans, mannans or glycoproteins between yeasts and hyphae have been described to induce distinct profiles of cytokine responses [9]. Along this line, we showed that stimulation with yeast or hyphae led to different metabolic responses in monocytes. Heat-killed *C*. *albicans* yeast induced production of proinflammatory cytokines, through a process highly demanding for the cells, which makes use of glycolysis, oxidative phosphorylation and glutaminolysis in order to fulfill the expensive energy requirements. In the case of heat-killed *C*. *albicans* hyphae, cytokine production was solely dependent on glycolysis. The transition to hyphal growth creates the opportunity for improved recognition of inner cell wall components such as β–glucan, which become more exposed, allowing their interaction with PRRs and triggering the proinflammatory cytokine production [18]. The data presented here, correlates with previously published data showing a strong induction of glycolysis by β–glucan [19] and argues for a model in which the poor β–glucan recognition in yeasts [18] requires different recognition systems for activation of multiple metabolic pathways, while the broad exposure of β–glucan by hyphae induces a much stronger induction of glycolysis in the immune cells which is sufficient to fulfill their energy requirements.

Glucose that enters the cell is not solely processed via glycolysis, but can be also transformed into fatty acids, aldoses, glycogen, or enter the pentose phosphate pathway to generate NADPH and pentoses [34]. ROS production has been historically linked to NADPH oxidase and mitochondrial activity [35], and several studies have assigned an important role for ROS production in host defense against microbial infections [29,36]. In our study, we found that glucose metabolism is crucial for the generation of ROS in human monocytes after *C*. *albicans* stimulation. Nonetheless, this could not be only attributed to glycolysis, as the impairment of the pentose phosphate pathway also induced a severe impairment of ROS production, as similarly described for LPS-activated macrophages, where ROS were generated through pentose phosphate pathway-dependent NADPH-oxidase activity [29]. These data suggest a differential role for the different metabolic pathways triggered after *C*. *albicans* stimulation in monocytes. While glycolysis appeared to play a central role in cytokine and ROS production, other routes such as oxidative phosphorylation or glutaminolysis seemed to play a preferential role in fueling cytokine induction. In contrast, the pentose phosphate pathway, which did not play a role in cytokine production, seems fundamental for ROS generation. These results further emphasize the variety and the specificity of the metabolic changes that cells undergo after making contact with a pathogen, highlighting the necessity of studying the distinctive features of the various stimuli.

The presence of suitable carbon sources in the environment is fundamental not only for the host but also for *Candida* cells. Regarding this, some studies have shown that the exposure of *Candida* to high concentrations of lactate is able to modulate its cell wall architecture, drug resistance and virulence [37,38]. In our study, the concentrations of lactate reached after stimulation are much lower than those demonstrated to alter cell wall structure of *C*. *albicans*, being glucose the major carbon source in all the experimental conditions tested. Therefore, in this case we find improbable that any of the experimental conditions might have been affected by the minor presence of lactate in the medium, compared to glucose.

TLR-mediated immunometabolic reprogramming have been reviewed by a number of authors [39–41]. *C*. *albicans* expresses a large variety of structures in its cell wall, which represent PAMPs that bind to different families of PRRs on the immune cells, among which CLRs and TLRs are the most important [8]. Especially deficiencies in CLRs lead to an impaired cytokine production and higher susceptibility to *C*. *albicans* infections [42,43]. In line with this, blockade of recognition of fungal β-glucan and mannans by C-type lectins led to a decrease in the glycolytic activity of monocytes triggered after stimulation with heat-killed yeast or with live wild-type *C*. *albicans*. Of note, the different background of the strains used can contribute to the complexity of the interpretation of the results obtained in this work. Besides this, our results suggest that the impact of metabolism in the outcome of *Candida* infection is not strain-specific, as experiments with heat-killed stimuli and with live fungi were carried out with strains with different backgrounds (UC820 in case of heat-killed fungi and SC5314 in case of live fungi).

We validated *in vivo* results on the role of cellular metabolism for antifungal host defense in a mouse model of systemic *C*. *albicans* infection, in which glycolysis-mediated mechanisms were demonstrated to be crucial for the defense against the pathogen. Inhibition of glycolysis led to hosts that were significantly more susceptible to the infection, presenting lower fungicidal activity and defective cytokine production capacity. Our data also suggest that glutamine metabolism could play a role in the production of IL-1β and its known downstream target IL-6 [44] after *C*. *albicans* recognition both in human and mouse. The relationship between IL-1β and IL-6 could be also playing a role in the effects observed on Th1 and Th17-derived cytokines in human PBMC cultures, whose decrease could be due to direct effects of the pharmacological inhibitors employed or to indirect effects of the dampening of IL-1β and IL-6 released from monocytes. Our results reflect the concept that the inhibition of glucose metabolism during *C*. *albicans* infection has an impact on the immune system at different levels, as the impairment of glycolysis decreased the ability to fight candidiasis both by direct and indirect mechanisms, as previously described for NK cells in an *in vitro* study [45]. In this sense, treatment with 2-DG induced a decrease in the production of monocyte-derived cytokines proved crucial to boost *Candida* clearance by neutrophils such as IL-1β, IL-6 and TNFα[8,46,47] and also reduced the fungicidal potential of neutrophils directly treated with this metabolic inhibitor. These results suggest that the establishment of a functional response against systemic *C*. *albicans* infections *in vivo* largely relies on the proper functioning of glucose metabolism in different immune subsets, demonstrating the importance of glycolysis in the development of an efficient antifungal response and highlighting the central role of metabolism as a cornerstone of the immune function.

The different pathways of the cellular metabolism are connected through a very complex network of enzymes and mediators. Our findings suggest that the immune functions of monocytes in *C*. *albicans* infection rely on the activation of glucose metabolism, but also required the participation of other additional metabolic pathways such as glutaminolysis or the pentose phosphate pathway. These results also delve into the distinctive features of CLR-mediated antifungal responses and highlight the need of studying the particular characteristics of the metabolic mechanisms underlying the immune responses against the wide variety of human pathogens. As combining effective anti-fungal treatment with adjuvant immunotherapy is proposed to improve the poor outcome in disseminated *C*. *albicans* infections, these data suggest that cellular metabolism of immune cells may represent a novel potential therapeutic target.

## Materials and methods

### Peripheral blood mononuclear cell and monocyte isolation

Buffy coats from healthy donors were obtained after written informed consent (Sanquin Blood Bank, Nijmegen, the Netherlands). Samples were anonymized to safeguard donor privacy. The use of the samples received IRB approval. Peripheral blood mononuclear cell (PBMC) isolation was performed by dilution of blood in pyrogen-free PBS and differential density centrifugation over Ficoll-Paque (GE Healthcare). Cells were washed three times in PBS. Percoll isolation of monocytes was performed as previously described (Repnik et al., 2003). Briefly, 150–200 x 10^6^ PBMCs were layered on top of a hyper-osmotic Percoll solution (48.5% Percoll [Sigma-Aldrich], 41.5% sterile H2O, and 0.16 M filter-sterilized NaCl) and centrifuged for 15 min at 580g. The interphase layer was isolated and cells were washed with cold PBS. Cells were re-suspended in RPMI culture medium (RPMI medium Dutch modified, Invitrogen) supplemented with 50 μg/mL gentamicin, 2 mM Glutamax, and 1 mM pyruvate, and counted. An extra purification step was added by adhering Percoll-isolated monocytes to polystyrene flat bottom plates (Corning) for 1 h at 37°C; a washing step with warm PBS was then performed to yield maximal purity.

### *Candida* strains and growth conditions

*Candida albicans* UC820 (ATCC MYA-3573) [48] was grown overnight to generate yeast cells in Sabouraud dextrose broth at 29°C, with shaking. Cells were harvested by centrifugation, washed twice with PBS, and re-suspended in culture medium (RPMI 1640 Dutch modification). To generate hyphae, yeast cells were inoculated and grown overnight at 37°C in culture medium adjusted to pH 6.4 with hydrochloric acid. *C*. *albicans* yeast or hyphae were heat-killed for 30 min at 95°C. The hyphae-specific G_1_ cyclin-null *hgc1*Δ mutant and *hgc* wild-type strains, kindly provided by Dr. Yue Wang (Institute of Molecular and Cell Biology, Singapore [49]) were grown under similar conditions.

### Transcriptome analysis

PBMCs at 5 x 10^6^ cells/mL were stimulated for 4 or 24 h with RPMI or 10^5^ heat-killed *C*. *albicans* yeast. Global gene expression was profiled using Illumina Human HT-12 Expression BeadChip according to manufacturer’s instructions. Image analysis, bead-level processing and quantile normalization of array data were performed using the Illumina LIMS platform, BeadStudio. Only samples that had cells for which paired fold-changes could be calculated were used (i.e. for which cells from the same individual were used for both RPMI and *Candida* stimulation). At 4 h there were 19 matched samples, and at 24 h there were 29 matched samples. Log_2_ fold changes (gene expression after *Candida* divided by expression after RPMI stimulation) were calculated for each individual separately. The average of these values was used in the plot. Gene identifiers were mapped to *entrez* ids, and formatted to match the transcript gene identifiers as defined in “RECON1”, the model that contains the enzymes associated with each reaction. Gene expression values were always mapped to alternative transcript 1, i.e. all *entrez* identifiers were appended with “_AT1”.

The tool “Escher” [50] was used to generate a pathway to visualize the expression data containing the most interesting parts of it.

### Inhibition experiments

100 μL monocytes at 1 x 10^6^ cells/mL or PBMCs at 5 x 10^6^ cells/mL were added to flat-bottom or round-bottom 96-well plates (Greiner), respectively. Cells were incubated with culture medium with 10% serum only as a negative control or incubated with 11 mM 2-deoxyglucose (Sigma), 100 nM Torin1 (Tocris), 1 mM AICAR (5-aminoimidazole-4-carboxamide-1-β-D-ribofuranoside, Brunschwig Chemie, Amsterdam, The Netherlands), 50 nM BPTES (Sigma), 3 μM potassium dichloroacetate (DCA, Sigma), 500 nM 6-aminonicotinamide (6-AN, Sigma), 10 μM etomoxir (Sigma), 1 μM oligomycin (Sigma). After 1 h, cells were stimulated with 10^5^ heat-killed *Candida* yeast, 10^5^ heat-killed *Candida* hyphae, 10^4^ live *hgc1 Candida* yeast or 10^4^ live *Δhgc1 Candida* yeast. The different concentrations of *Candida* used, were based on optimization experiments and employed as seen in works from different groups [51–53]. Similar concentrations of yeasts and hyphae were employed at similar concentrations in order to get a better approach to *in vivo* situations. Supernatants from monocytes were collected 24 h after stimulation. Supernatants from PBMCs were collected 7 days after stimulation, except for IL-10 production, which was measured after 48 h. Concentrations of inhibitors were selected as being the highest non-cytotoxic concentrations (see S8 Fig). All supernatants were stored at -20°C until analyzed.

For receptor blockade experiments, before stimulation with *C*. *albicans*, monocytes were preincubated for 1 h with 100 μg/mL laminarin (Sigma), 10 μg/mL anti-CR3 antibody and control IgG (R&D), 100 ng/mL *B*. *quintana* LPS, 10 μg/mL TLR2-blocking antibody (anti-TLR2) and its control IgA1 (InvivoGen, San Diego, CA), 5 μg/mL MR-blocking antibody (anti-MR, R&D) and IgG1 isotype control (BD Biosciences).

### Cytokine measurements

Cytokine production from human cells was determined in supernatants using commercial ELISA kits for IL-1β, TNFα, IL-17A, IL-22 (R&D Systems, Minneapolis, MN) IL-6, IFNγ, and IL-10 (Sanquin, Amsterdam, The Netherlands), following the instructions of the manufacturer.

### Metabolite measurements

Lactate was measured from cell culture supernatants using a coupled enzymatic assay in which lactate was oxidized and the resulting H_2_O_2_ was coupled to the conversion of Amplex Red reagent to fluorescent resorufin by HRP (horseradish peroxidase) [54]. Glucose consumption was measured according to the manufacturer instructions using the Amplex Red Glucose/Glucose Oxidase Assay Kit (Life Technologies). Glutamate, fumarate, malate, α-ketoglutarate and succinate concentrations were determined by commercial assay kits (all Sigma) following the instructions of the manufacturer from at least one million monocytes lysed in 1 mL 0.5% Triton-X in PBS at 4 and 24 h after stimulation.

### Assessment of oxygen consumption and acidification rates

Real-time analysis of ECAR, OCR and SRC on monocytes was performed using an XF-96 Extracellular Flux Analyzer (Seahorse Bioscience) as described in Lachmandas et al., 2016. CD14^+^ monocytes were purified from freshly isolated PBMCs using MACS microbeads for positive selection, according to the manufacturer’s instructions (Miltenyi Biotec). These monocytes were seeded in quintuplicate in XF-96 cell culture plates (2 × 10^5^ monocytes/well) in the presence of RPMI or *C*. *albicans* for 4h or 24 h in 10% human pooled serum. For the measurements of oxygen consumption and acidification rates it is therefore important to have a homogenous cell population, in this case monocytes. The CD14^+^ isolation is performed on PBMCs, which contain usually less than 5% of neutrophils. With subsequent CD14^+^ selection we obtain 95% of monocytes, so the amount of neutrophils and lymphocytes in the CD14^+^ selected cells is negligible. The metabolic rates of monocytes were analyzed in four consecutive measurements in XF Base Medium (unbuffered DMEM with 5.5 mM glucose and 2 mM L-glutamine, pH adjusted to 7.4). After three basal measurements, three consecutive measurements were taken following the addition of 1.5 μM oligomycin, 1 μM carbonyl cyanide-4-(trifluoromethoxy) phenylhydrazone (FCCP), 2 μM antimycin together with 1 μM rotenone, glucose (20 mM), pyruvate (1 mM) and/or 50 mM 2-DG in order to determine basal and maximum OCR and ECAR. SRC was determined as the absolute increase in OCR after FCCP injection compared with basal OCR. All compounds used during the Seahorse runs were acquired from Sigma-Aldrich.

### mRNA extraction and RT-PCR

Cells were cultured as described above. After 4 h and 24 h mRNA was extracted by TRIzol (Life Technologies), according to the manufacturer’s instructions, and cDNA was synthesized using iScript reverse transcriptase (Invitrogen). Relative mRNA levels were determined using the Applied Biosciences StepOne PLUS and the SYBR Green method (Invitrogen). Values are expressed as fold increases in mRNA levels, relative to those in non-stimulated cells, with HPRT as housekeeping gene. Primers are listed in S1 Table.

### Reactive oxygen species (ROS) assay

Oxygen radical production levels of isolated monocytes were evaluated using luminol-enhanced chemiluminescence and determined in an automated LB96V Microlumat plus luminometer (EG & G Berthold, Bald Wilberg, Germany) as previously described [55]. Briefly, monocytes (1 × 10^5^ per well) were seeded into 96-well plates and incubated in medium containing either RPMI, phorbol 12-myristate 13-acetate (PMA; 5 μg/ml), heat-killed *C*. *albicans* yeast or heat-killed *C*. *albicans* hyphae (10^7^ CFU/ml). Luminol was added to each well in order to start the chemiluminescence reaction. Each measurement was carried out in at least duplicate repetitions. Chemiluminescence was determined every 145 s at 37°C for 1 h. Luminescence was expressed as relative light units (RLU) per second.

### Ethics statement

Monocytes and PBMCs were isolated from blood donated by healthy volunteers after written informed consent. Ethical approval was obtained from the CMO Arnhem-Nijmegen (NL32357.091.10). Buffy coats from healthy donors were obtained after written informed consent (Sanquin Blood Bank, Nijmegen, the Netherlands). Samples were anonymized to safeguard donor privacy. The use of the samples received IRB approval.

All animal work was approved by the Animal Care and Use Committee of the Centro Nacional de Biotecnología—CSIC (protocol number 312–2014) in accordance with Spanish RD 1201/2005 and international EU guidelines 2010/63/UE about protection of animals used for experimentation and other scientific purposes and Spanish national law 32/2007 about animal welfare in their exploitation, transport and sacrifice.

### *In vivo Candida* infection assays

8–12 week-C57BL/6J mice were randomized and treated with a daily intraperitoneal dose of 100 mg/kg 2-deoxyglucose (n = 6) or 100 μg BPTES (n = 6) every morning for 5 days starting at the same day with the *C*. *albicans* intravenous infection. PBS was injected as a control (n = 6). *C*. *albicans* SC5314 yeast were grown on YPD plates (Sigma-Aldrich, St Louis, MO) at 30°C for 48 h. Then, *C*. *albicans* cells were centrifuged, washed in PBS and counted using a hematocytometer. Mice were infected by intravenous injection of 1 x 10^5^ *C*. *albicans* yeast via the lateral tail vein and daily monitored for health and survival following the institutional guidance. After 5 days, mice were euthanized in a CO_2_ rodent euthanasia chamber and kidneys were aseptically removed, weighed and homogenized in PBS using a T10 basic Ultra-Turrax homogenizer (Ika, Staufen, Germany). Fungal burden was determined by plating organ homogenates in serial dilutions on YPD plates. Colony forming units (CFUs) were counted after growth for 48 h at 30°C.

### Analysis of cytokine production by mouse splenocytes

For *ex vivo* stimulation experiments splenocytes were obtained from mice at day 5 of i.v. infection with *C*. *albicans* and stimulated *ex vivo* with LPS (10 ng/mL) heat-killed *Candida* yeast (1 × 10^7^/mL) or heat-killed *C*. *albicans* hyphae (1 × 10^6^/mL). Splenocytes were obtained by gently squeezing spleens in a sterile 100 mm filter. After centrifugation and washing, splenocytes were resuspended in complete RPMI 1640 medium supplemented with 10% FCS, 2 mM L-glutamine, 100 U/mL penicillin, 100 μg/mL streptomycin and 50 μM 2-mercaptoethanol, and counted using a hematocytometer. Splenocyte concentration was adjusted to 5 × 10^6^/mL. 200 μL of the cell suspension were cultured in round-bottom 96-well plates (Corning, Durham, NC) and stimulated with RPMI or 1 × 10^6^ heat-killed *C*. *albicans* yeast or hyphae/mL. The measurement of cytokine concentrations was performed in supernatants collected after 48 h of incubation at 37°C in 5% CO_2._ Cytokine production from mouse cells was determined in supernatants using commercial ELISA kits for IL-1β, TNFα, IL-6, IFNγ and IL-10, all from BD Pharmingen (San Diego, CA).

### Neutrophil killing assays

Circulating neutrophils were isolated from blood drawn by cardiac puncture, diluted in PBS containing 5 mM EDTA and 3% FCS, overlaid over a density gradient of Histopaque 1119 and Histopaque 1077 (Sigma) and centrifuged for 30 minutes at 400 g. Neutrophil preparations had a purity >80%. To test neutrophil killing activity, 5 x 10^4^ *C*. *albicans* yeast were exposed to 10^4^ neutrophils for 2 h; neutrophils were then lysed with water and the number of surviving yeast cells was assessed on YPD agar. Killing activity was expressed as the percentage of *C*. *albicans* cells surviving in the presence of neutrophils compared to *C*. *albicans* cells surviving in the absence of neutrophils.

### Phagocytosis assays

Percoll monocytes were plated in 96 flat bottom plates at 1x10^5^ cells / well. Cells were allowed to phagocytose 1 x 10^6^ (MOI 1:10) heat inactivated FITC-labeled *C*. *albicans* for 2h in the presence or absence of α-antitrypsin 10 mg/mL or 100 mg/mL. Subsequently, the fluorescence signal of extracellular non-phagocytosed *Candida* was quenched using trypan blue. The monocytes that phagocytosed one or more *C*. *albicans* yeast were enumerated by their positivity for the FITC signal, and could be divided into two populations: FITC^-^ monocytes (those that did not engulf *C*. *albicans*) and FITC^+^ monocytes (those that did).

### Viability assays

Cell viability was assessed using Annexin-V (Biovision, San Francisco, CA) and propidium iodide (Sigma) staining. Cells were stained for 15 minutes using Annexin-V-FITC using the protocol supplied by the manufacturer to detect early apoptotic cells. Subsequently cells were stained with for 5 minutes in 10 ug/mL propidium iodide. Cells were assessed for annexin-V and PI positivity using a FC500 flow cytometer (Beckman Coulter). Annexin-V^+^ cells were considered as early apoptotic cells and Annexin-V^+^ PI^+^ cells were considered as late apoptotic cells.

## Supporting information

S1 Fig Transcriptome analysis after stimulation of PBMCs with *C*. *albicans*. (A and B) Pathway map of the gene expression in the main metabolic pathways in PBMCs stimulated with heat-killed *C*. *albicans* yeast 4 h (A) and 24 h (B) after the stimulation. The transcripts marked in red were significantly upregulated in *C*. *albicans* versus RPMI.
(TIF)

S2 Fig OCR/ECAR ratios. Basal and maximum OCR/ECAR ratios obtained after 4 h and 24 h stimulation of monocytes with medium or heat-killed *C*. *albicans* yeast (mean ± SEM, n = 6–8; pooled from 2 independent experiments).
(TIF)

S3 Fig Glycolysis, glutaminolysis and oxidative phosphorylation differentially affected Th1/Th17-derived cytokine production in PBMCs. (A-B) IL-17, IL-22 and IFNγ production by human PBMCs treated with different metabolic inhibitors and stimulated with heat-killed *C*. *albicans* yeast (A) or heat-killed *C*. *albicans* hyphae (B) for 7 days. IL-10 production was measured after 48 h of culture. (mean ± SEM, n = 6; pooled from 2 independent experiments). *p<0.05, Wilcoxon signed-rank test.
(TIF)

S4 Fig C-type lectins triggered glycolysis after stimulation with *Candida* yeast. (A-B) Lactate production by human monocytes was measured after adding the corresponding isotype controls of the blockers used in Fig 4 and the subsequent 24 h-stimulation with medium, heat-killed *C*. *albicans* yeast or heat-killed *C*. *albicans* hyphae (A) or *hgc1* or *Δhgc1* live *C*. *albicans* (mean ± SEM, n = 6; pooled from 2 independent experiments). isoCR3: IgG; isoTLR2: anti-IgA1; isoMR: IgG1. Wilcoxon signed-rank test.
(TIF)

S5 Fig ROS production by monocytes involved glycolysis and the pentose phosphate pathway. (A-D) Human monocytes were treated with DMSO (A), Torin1 (B), AICAR (C) or BPTES (D) and subsequently stimulated with medium, heat-killed *C*. *albicans* yeast or heat-killed *C*. *albicans* hyphae. Luminescence generated from ROS production was measured every 145 seconds during 60 minutes (n = 4; pooled from 2 independent experiments).
(TIF)

S6 Fig Phagocytic capacity of monocytes after 2-DG treatment. (mean ± SEM, n = 12; pooled from 4 independent experiments).
(TIF)

S7 Fig Direct effects of 2-DG on mouse neutrophils. (A-B) Candidacidal activity of neutrophils isolated from blood of non-infected C57BL/6 mice following the protocol described in (A) after *in vitro* treatment of cells with PBS or 11 mM 2-DG (mean ± SEM, n = 6). *p< 0.05, Student’s t test. Each dot represents one mouse.
(TIF)

S8 Fig Viability of human PBMCs treated with metabolic inhibitors. PBMCs were stained for Annexin V and propidium iodide. Annexin V^+^ cells were considered as early apoptotic cells and Annexin V^+^ / PI^+^ cells were considered as late apoptotic cells. (mean ± SEM, n = 3). Similar results were obtained in 3 independent experiments.
(TIF)

S1 Table Primers for real-time PCR. (DOCX)
