# Supplementary figures and images for: Rewiring monocyte glucose metabolism via C-type lectin signaling protects against disseminated candidiasis
Source: PLoS Pathog. 2017 Sep 18;13(9):e1006632. doi: 10.1371/journal.ppat.1006632 (PMC5619837; doi:10.1371/journal.ppat.1006632)

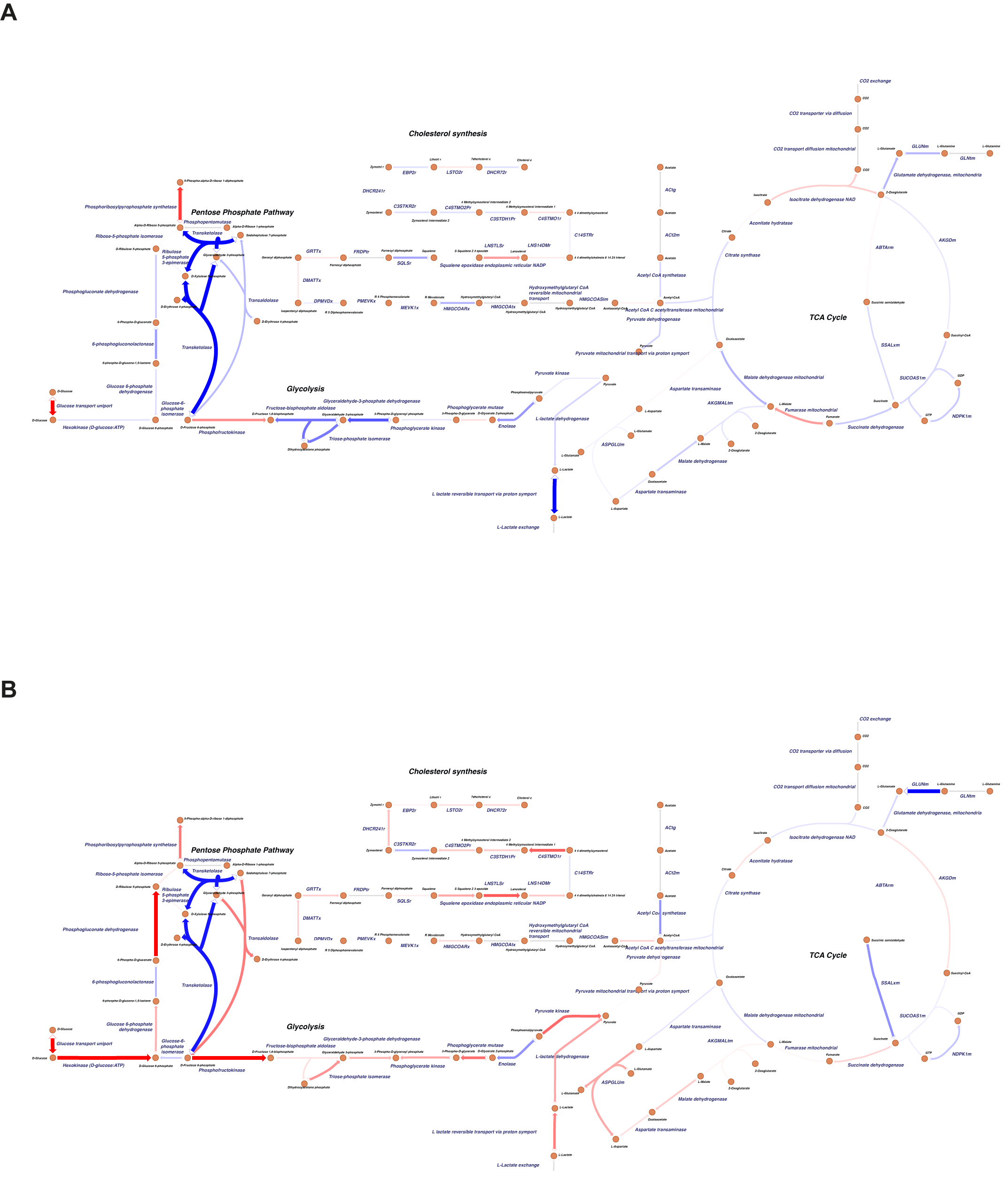

Supplement: S1 Fig — (A and B) Pathway map of the gene expression in the main metabolic pathways in PBMCs stimulated with heat-killed C. albicans yeast 4 h (A) and 24 h (B) after the stimulation. The transcripts marked in red were significantly upregulated in C. albicans versus RPMI. (TIF) [file ppat.1006632.s001.tif]

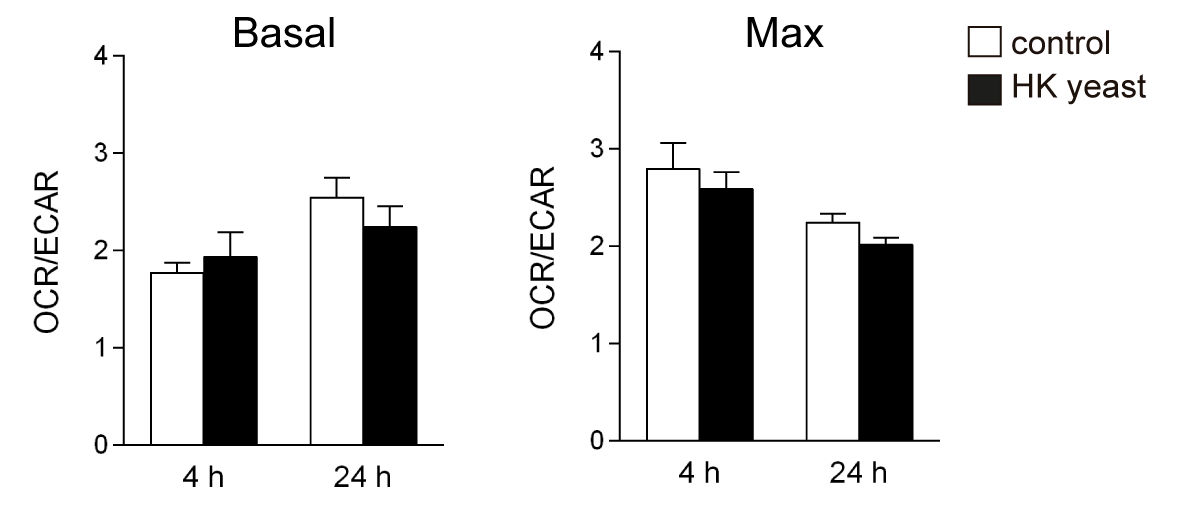

Supplement: S2 Fig — Basal and maximum OCR/ECAR ratios obtained after 4 h and 24 h stimulation of monocytes with medium or heat-killed C. albicans yeast (mean ± SEM, n = 6–8; pooled from 2 independent experiments). (TIF) [file ppat.1006632.s002.tif]

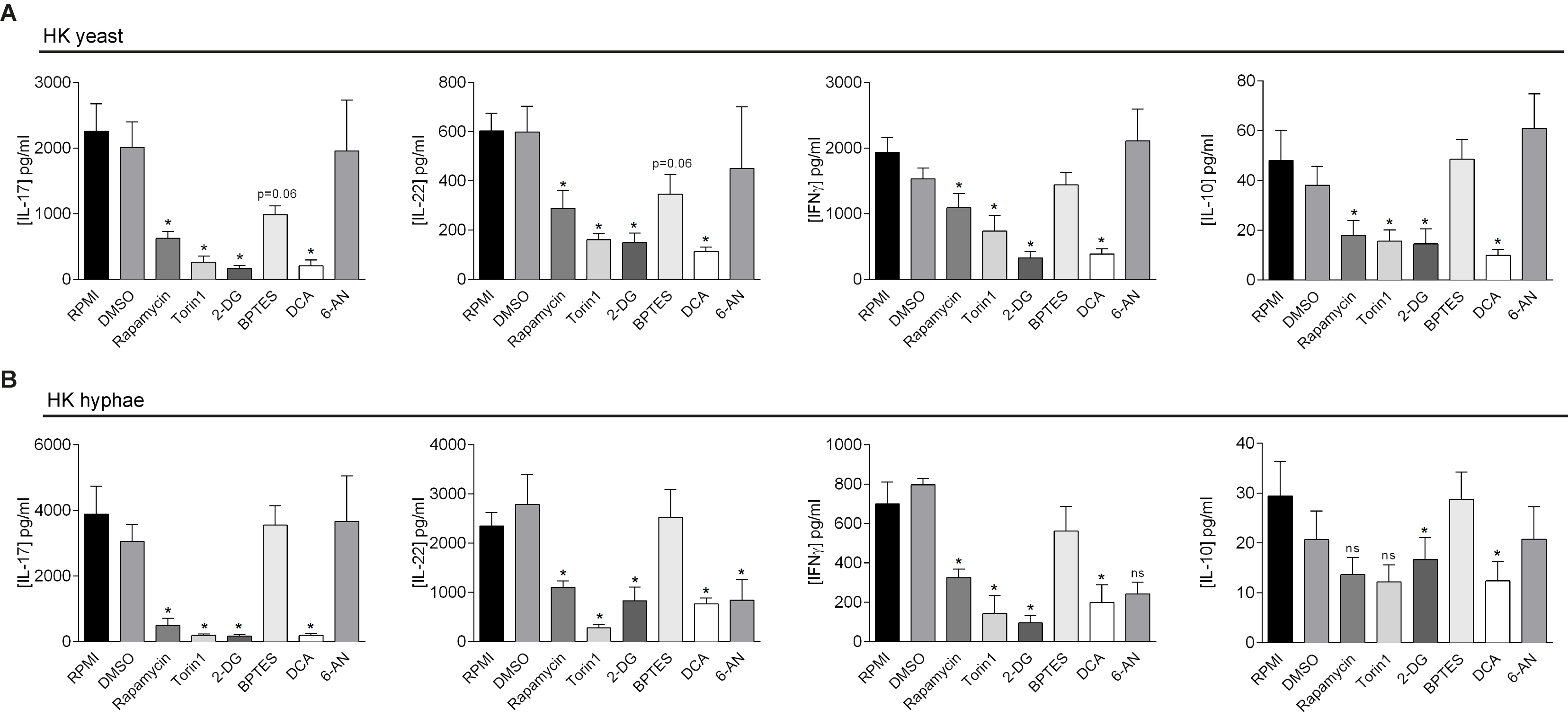

Supplement: S3 Fig — (A-B) IL-17, IL-22 and IFNγ production by human PBMCs treated with different metabolic inhibitors and stimulated with heat-killed C. albicans yeast (A) or heat-killed C. albicans hyphae (B) for 7 days. IL-10 production was measured after 48 h of culture. (mean ± SEM, n = 6; pooled from 2 independent experiments). *p<0.05, Wilcoxon signed-rank test. (TIF) [file ppat.1006632.s003.tif]

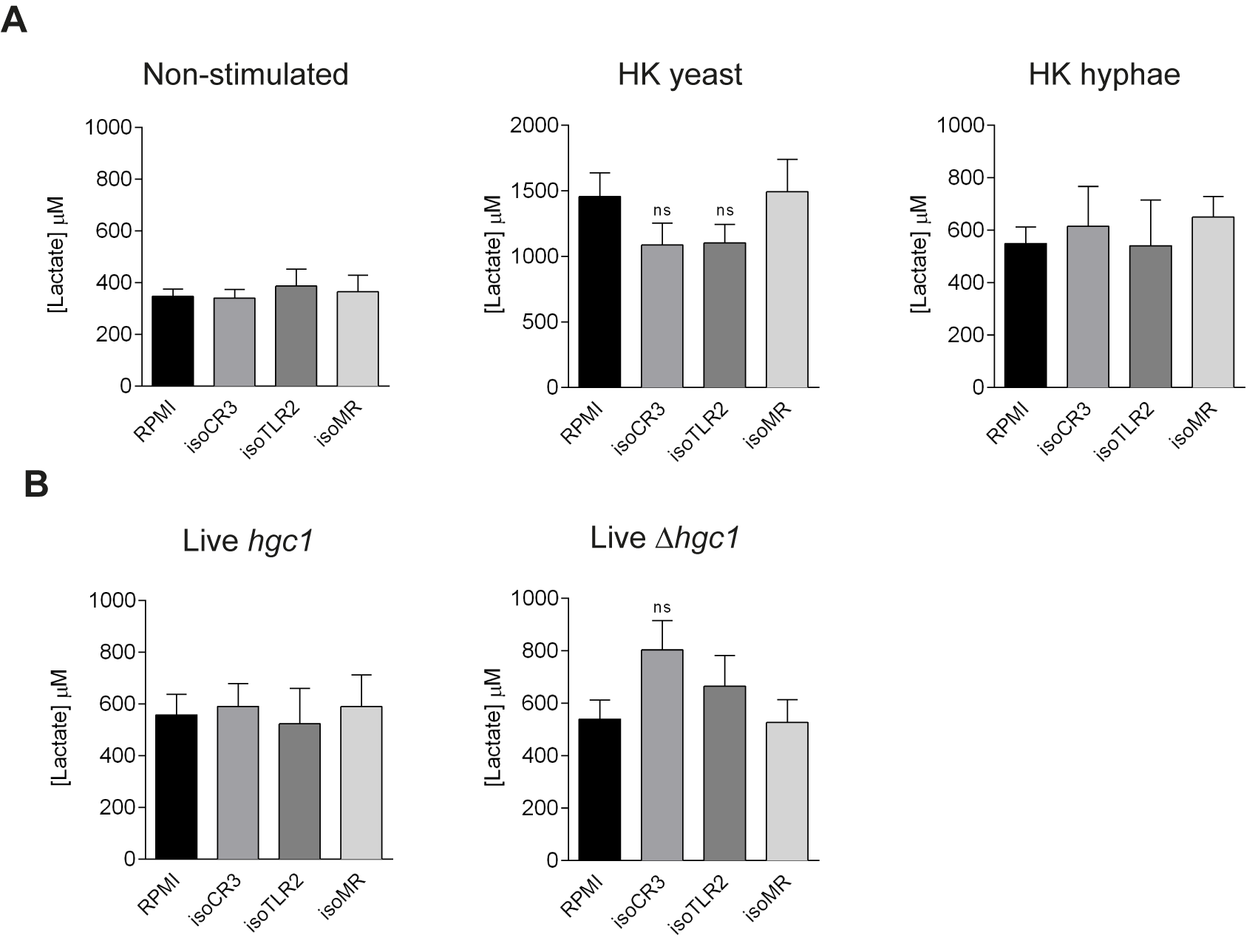

Supplement: S4 Fig — (A-B) Lactate production by human monocytes was measured after adding the corresponding isotype controls of the blockers used in Fig 4 and the subsequent 24 h-stimulation with medium, heat-killed C. albicans yeast or heat-killed C. albicans hyphae (A) or hgc1 or Δhgc1 live C. albicans (mean ± SEM, n = 6; pooled from 2 independent experiments). isoCR3: IgG; isoTLR2: anti-IgA1; isoMR: IgG1. Wilcoxon signed-rank test. (TIF) [file ppat.1006632.s004.tif]

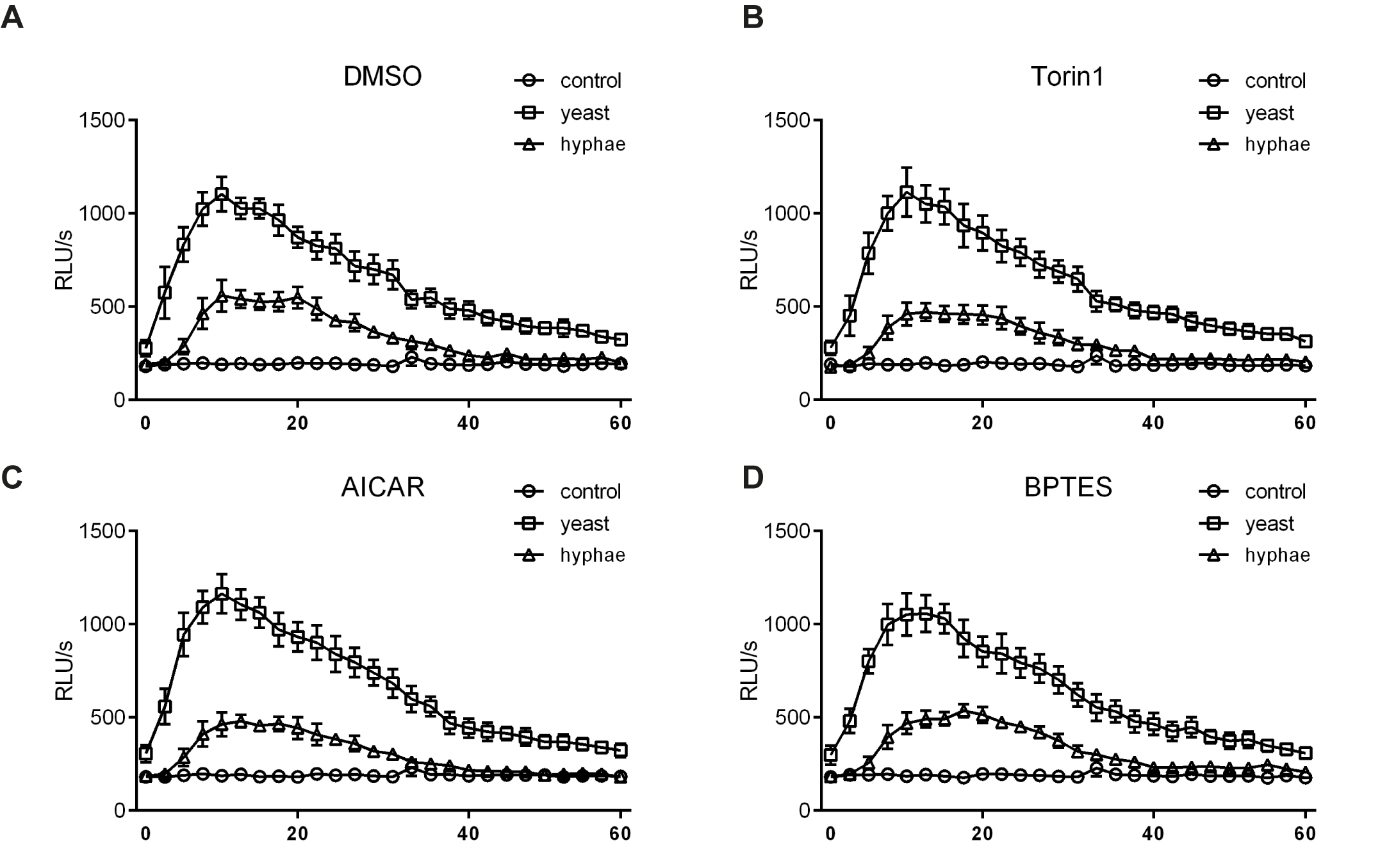

Supplement: S5 Fig — (A-D) Human monocytes were treated with DMSO (A), Torin1 (B), AICAR (C) or BPTES (D) and subsequently stimulated with medium, heat-killed C. albicans yeast or heat-killed C. albicans hyphae. Luminescence generated from ROS production was measured every 145 seconds during 60 minutes (n = 4; pooled from 2 independent experiments). (TIF) [file ppat.1006632.s005.tif]

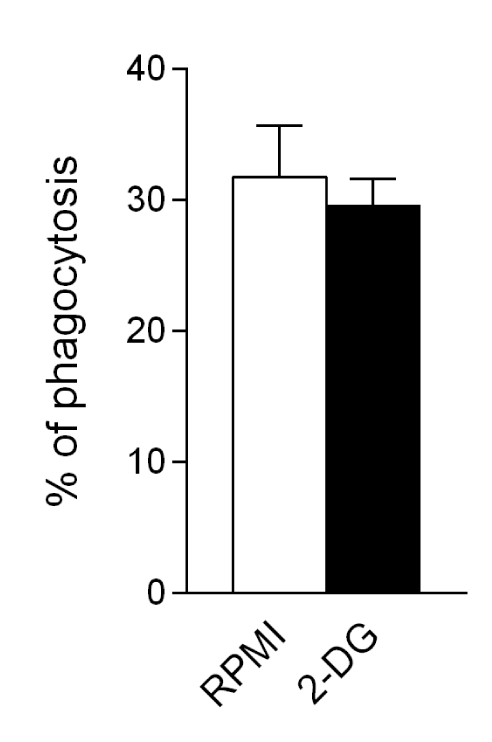

Supplement: S6 Fig — (mean ± SEM, n = 12; pooled from 4 independent experiments). (TIF) [file ppat.1006632.s006.tif]

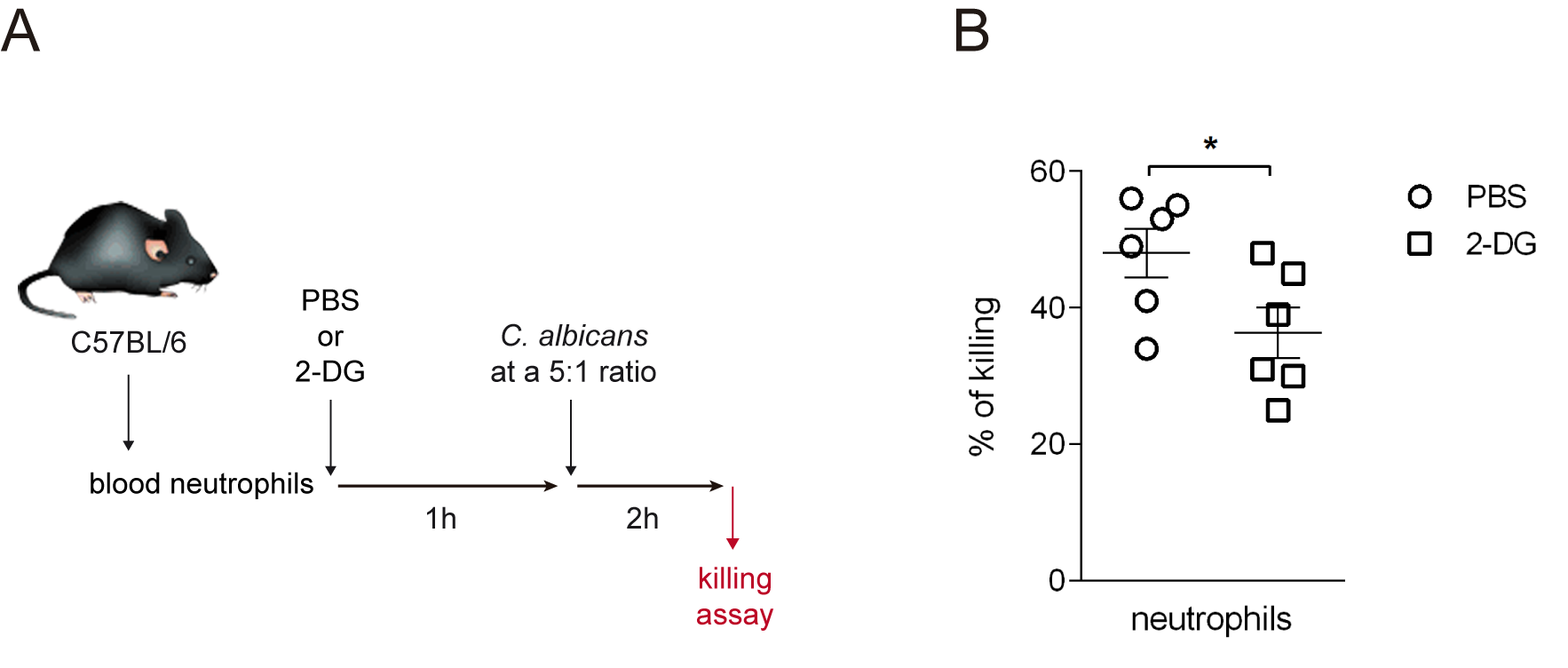

Supplement: S7 Fig — (A-B) Candidacidal activity of neutrophils isolated from blood of non-infected C57BL/6 mice following the protocol described in (A) after in vitro treatment of cells with PBS or 11 mM 2-DG (mean ± SEM, n = 6). *p< 0.05, Student’s t test. Each dot represents one mouse. (TIF) [file ppat.1006632.s007.tif]

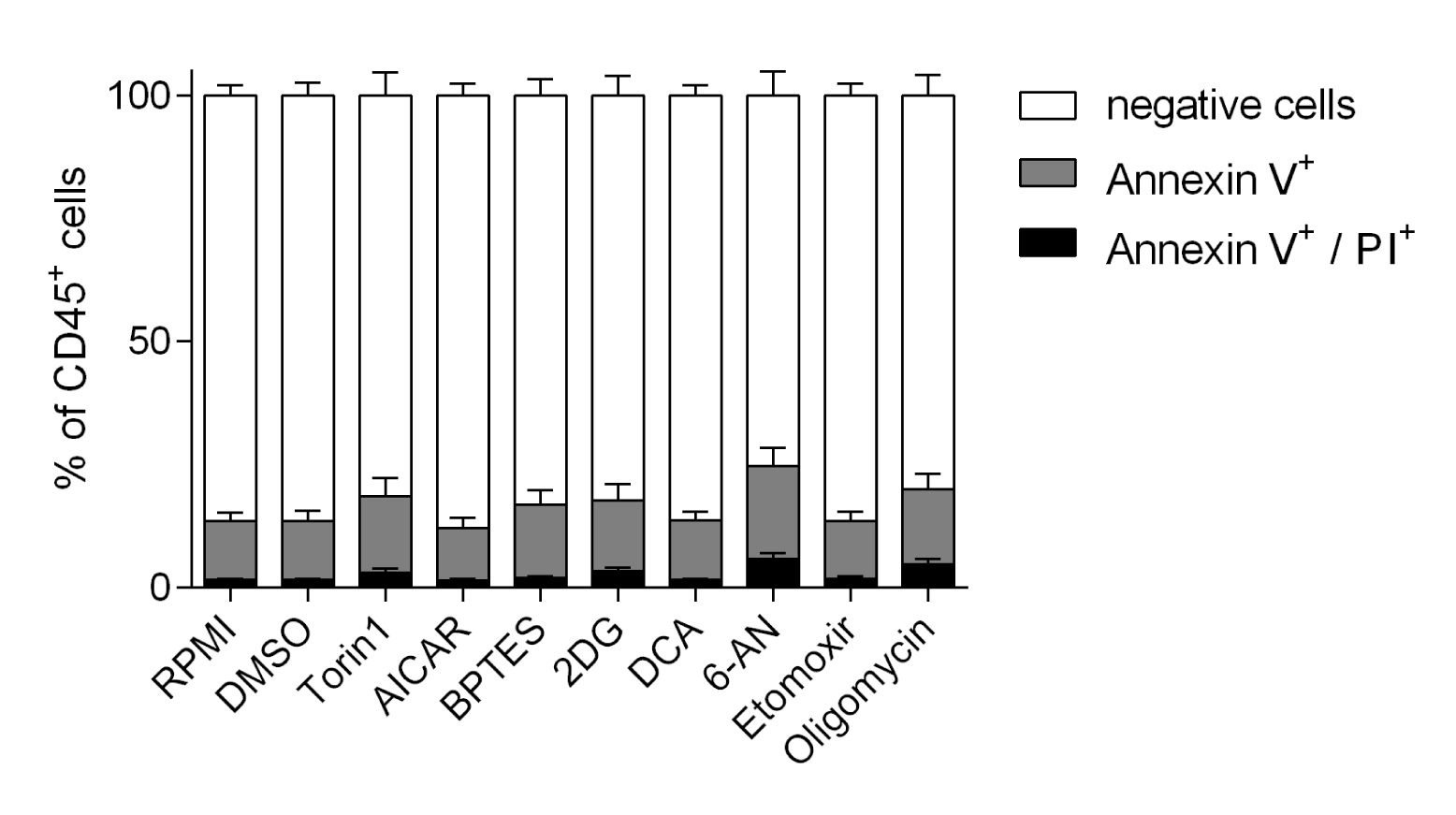

Supplement: S8 Fig — PBMCs were stained for Annexin V and propidium iodide. Annexin V+ cells were considered as early apoptotic cells and Annexin V+ / PI+ cells were considered as late apoptotic cells. (mean ± SEM, n = 3). Similar results were obtained in 3 independent experiments. (TIF) [file ppat.1006632.s008.tif]
