# Supplementary material for: Rewiring monocyte glucose metabolism via C-type lectin signaling protects against disseminated candidiasis
Source: PLoS Pathog. 2017 Sep 18;13(9):e1006632. doi: 10.1371/journal.ppat.1006632 (PMC5619837; doi:10.1371/journal.ppat.1006632)
Supplement: S1 Table — (DOCX) [file ppat.1006632.s009.docx]

**Table S1. Primers for Real-Time PCR**

| Gene | Primer | Sequence 5’-3’ |
| --- | --- | --- |
| HPRT | HPRT-for | CCTGGCGTCGTGATTAGTGAT |
|  | HPRT-rev | AGACGTTCAGTCCTGTCCATAA |
| MTOR | MTOR-for | TCCGAGAGATGAGTCAAGAGG |
|  | MTOR-rev | CACCTTCCACTCCTATGAGGC |
| HK2 | HK2-for | TTGACCAGGAGATTGACATGGG |
|  | HK2-rev | CAACCGCATCAGGACCTCA |
| PFKP | PFKP-for | ATTGCGGTTTTCGATGCCAC |
|  | PFKP-rev | GCCACAACTGTAGGGTCGT |
| GLS | GLS-for | AGGGTCTGTTACCTAGCTT |
|  | GLS-rev | ACGTTCGCAATCCTGTAGA |
| GLUD | GLUD-for | TCGTGGAGGACAAGTTGGT |
|  | GLUD-rev | TTGCAGGGCTTGATGATCC |
| α-KGDH | α-KGDH-for | TGCCAGCATATTGGGGTGG |
|  | α-KGDH-rev | GGAACTCCTCAAACCTGGTGG |
| LDH | LDH-for | ATGGCAACTCTAAAGGATCAGC |
|  | LDH-rev | CCAACCCCAACAACTGTAATCT |
